# Home Telemonitoring and a Diagnostic Algorithm in the Management of Heart Failure in the Netherlands: Cost-effectiveness Analysis

**DOI:** 10.2196/31302

**Published:** 2022-08-04

**Authors:** Fernando Albuquerque de Almeida, Isaac Corro Ramos, Maiwenn Al, Maureen Rutten-van Mölken

**Affiliations:** 1 Erasmus School of Health Policy and Management Erasmus University Rotterdam Rotterdam Netherlands; 2 Institute for Medical Technology Assessment Erasmus University Rotterdam Rotterdam Netherlands

**Keywords:** discrete event simulation, cost-effectiveness, early warning systems, home telemonitoring, diagnostic algorithm, heart failure

## Abstract

**Background:**

Heart failure is a major health concern associated with significant morbidity, mortality, and reduced quality of life in patients. Home telemonitoring (HTM) facilitates frequent or continuous assessment of disease signs and symptoms, and it has shown to improve compliance by involving patients in their own care and prevent emergency admissions by facilitating early detection of clinically significant changes. Diagnostic algorithms (DAs) are predictive mathematical relationships that make use of a wide range of collected data for calculating the likelihood of a particular event and use this output for prioritizing patients with regard to their treatment.

**Objective:**

This study aims to assess the cost-effectiveness of HTM and a DA in the management of heart failure in the Netherlands. Three interventions were analyzed: usual care, HTM, and HTM plus a DA.

**Methods:**

A previously published discrete event simulation model was used. The base-case analysis was performed according to the Dutch guidelines for economic evaluation. Sensitivity, scenario, and value of information analyses were performed. Particular attention was given to the cost-effectiveness of the DA at various levels of diagnostic accuracy of event prediction and to different patient subgroups.

**Results:**

HTM plus the DA extendedly dominates HTM alone, and it has a deterministic incremental cost-effectiveness ratio compared with usual care of €27,712 (currency conversion rate in purchasing power parity at the time of study: €1=US $1.29; further conversions are not applicable in cost-effectiveness terms) per quality-adjusted life year. The model showed robustness in the sensitivity and scenario analyses. HTM plus the DA had a 96.0% probability of being cost-effective at the appropriate €80,000 per quality-adjusted life year threshold. An optimal point for the threshold value for the alarm of the DA in terms of its cost-effectiveness was estimated. New York Heart Association class IV patients were the subgroup with the worst cost-effectiveness results versus usual care, while HTM plus the DA was found to be the most cost-effective for patients aged <65 years and for patients in New York Heart Association class I.

**Conclusions:**

Although the increased costs of adopting HTM plus the DA in the management of heart failure may seemingly be an additional strain on scarce health care resources, the results of this study demonstrate that, by increasing patient life expectancy by 1.28 years and reducing their hospitalization rate by 23% when compared with usual care, the use of this technology may be seen as an investment, as HTM plus the DA in its current form extendedly dominates HTM alone and is cost-effective compared with usual care at normally accepted thresholds in the Netherlands.

## Introduction

### Background

Heart failure is a major health concern associated with significant morbidity, mortality, and reduced quality of life in patients. An estimated 64.3 million people worldwide live with heart failure [1]. A meta-analysis based on echocardiographic screening studies in the general population in high-income countries revealed that the prevalence of heart failure is approximately 11.8% in those aged ≥65 years [2]. In 2019, the Dutch prevalence of heart failure was estimated to be 238,700, with an incidence of 37,400 new cases and 7264 deaths due to heart failure [3]. Accordingly, heart failure is responsible for elevated health care costs in the Netherlands: €817 million (currency conversion rate in purchasing power parity at the time of study: €1=US $1.29; further conversions are not applicable in cost-effectiveness terms) in 2017, corresponding to 8% of the costs for cardiovascular diseases and approximately 1% of the total health care expenditure for that year [4]. Of the total heart failure costs, 45% are attributable to care provided in the hospital and 43% are spent on care for older adults (long-term institutional older adult care, assisted-living facilities for older adults, and home care) [4].

Remote patient monitoring is a patient management approach that uses information and communication technologies to monitor and transmit physiological data related to patient health status between geographically separated individuals [5]. Home telemonitoring (HTM) is the particular case in which the monitoring and transmission of data are performed from the patient’s home. HTM facilitates frequent or continuous assessment of disease signs and symptoms, and it has shown to improve compliance by involving patients in their own care and prevent emergency admissions by facilitating early detection of clinically significant changes [6]. The use of information and communication technologies in the management of chronic diseases has become increasingly important, especially since the COVID-19 pandemic when routine care had to be postponed or replaced by remote alternatives. Evidence shows that HTM can have a positive impact on both mortality and hospital admissions [7-9], whereas other studies question the effectiveness [10] and cost-effectiveness [11] of home-based monitoring systems.

Diagnostic algorithms (DAs) can be defined as predictive mathematical relationships that make use of a wide range of collected data for calculating the likelihood of a particular event (eg, death or hospitalization). These algorithms use this output for prioritizing patients with regard to their treatment by raising alarms that trigger follow-up actions if the probability of the event exceeds a predefined threshold. Evidence shows that data-driven approaches looking at trends and patterns of change in recorded parameters improve the accuracy of detecting disease deterioration when compared with clinical decision rules [12-15]. Coupled with the fact that a large number of parameters associated with heart failure events can be measured with HTM, it is expected that advanced algorithms with better diagnostic performance will result in time efficiency when analyzing the data generated with HTM systems. Therefore, they may improve clinical decision-making by raising alerts in a manner that can be intuitively used by clinicians with a high degree of confidence [16]. However, health care funds are limited, and scarce resources must be allocated to patient subgroups for which new interventions are most beneficial.

### Objectives

The objective of this study was to assess the cost-effectiveness of HTM and a DA for the management of heart failure in the Netherlands. A base-case analysis was performed, and structural and parametric uncertainty was assessed through scenario, sensitivity, and value of information analyses. Furthermore, we focused particularly on the assessment of the cost-effectiveness of the DA at different levels of diagnostic accuracy of event prediction, that is, different points of its receiver operating characteristic (ROC) curve, and on the cost-effectiveness of the interventions under analysis for a wide range of patient subgroups.

## Methods

### Interventions

Three interventions were included in the cost-effectiveness analysis: (1) usual care; (2) HTM, as described in the Trans-European Network—Home-Care Management System (TEN-HMS) original publication [17] (HTM); and (3) HTM with the addition of a DA (HTM+DA).

Usual care consisted of an individualized written management plan by the investigator that described what pharmacological treatment patients should receive, in what order, and how it should be monitored. All patients required a loop diuretic according to the inclusion criteria. The management plan focused on the treatment of left ventricular systolic dysfunction with appropriate doses of angiotensin-converting enzyme inhibitors and β-blockers. If severe symptoms persisted, spironolactone was added to the therapeutic plan according to regional guidelines. Digoxin and anticoagulants were recommended for patients in atrial fibrillation. The patient management plan was sent to and implemented by the patient’s primary care physician [17].

HTM, as described in the original publication of the TEN-HMS [17], consisted of monitoring the patient’s weight, blood pressure, heart rate, and rhythm twice daily. Values greater than or less than the preset limits were notified automatically to the study nurses, who reviewed the information and took action either directly, for any short-term advice, or through the primary care physician, if long-term changes in therapy were required. Nurses could also manually scan patient data to identify any trends that they considered as requiring action. The study personnel were primarily responsible for the implementation of the management plan in patients assigned to HTM, whereas the primary care physician and the investigator were kept informed of all contacts.

HTM+DA consisted of a previously described HTM intervention with the addition of a DA published elsewhere [18]. The algorithm used data collected from patients with heart failure who adopted HTM as part of their daily health care. Their hospital records were retrospectively reviewed, and heart failure–related admissions data were collected. The DA used collected data (eg, blood pressure, heart rate, and weight) to predict patient hospitalization. The prediction or classification performance of the algorithm was assessed using an ROC analysis (curve shown in Figure S1 in Multimedia Appendix 1 [17-28]).

### Model Structure

The patient-level discrete event simulation model used for the analysis was developed and described in detail elsewhere [29]. Unlike other published health economic models for heart failure, this is a singular model that includes a wide range of patient characteristics and outcomes. The model consists of a series of regression equations describing the statistical associations between patient characteristics and changes in intermediate and final outcomes over time. The time-to-event regression equations were estimated using the patient-level data from the TEN-HMS study [17]. The model simulates the time to an outpatient visit, hospitalization, and death. Intermediate outcomes generated from the model are the number of outpatient visits, hospitalizations, and avoided hospitalizations. Final outcomes are the total life years, quality-adjusted life years (QALYs), and costs.

### Model Population

In the base-case analysis, patients were randomly sampled (with replacement) from the entire population included in the TEN-HMS study [17]. The baseline patient and disease characteristics of the study population are shown in Table 1. The presented patient and disease characteristics are a subset of the entire range of patient-level data available in the TEN-HMS study, and they represent the inputs used in the simulation. The patient population was assumed to be representative of the Dutch heart failure patient population.

Each patient was simulated for the three interventions included in the cost-effectiveness analysis: (1) usual care, (2) HTM, and (3) HTM + DA.

**Table 1:** Baseline patient and disease characteristics of the model population.

Baseline characteristics			Value
Sample size, n			426
EF^a^ (%), mean (SD)			25.06 (7.58)
Age (years), mean (SD)			67.56 (11.64)
SBP^b^ (mm Hg), mean (SD)			114.24 (19.25)
BMI (kg/m^2^), mean (SD)			26.17 (4.73)
Creatinine (µmol/L), mean (SD)			135.71 (51.98)
**NYHA^c^ class (%)**			
	1	79 (18.5)	
	2	185 (43.4)	
	3	132 (31)	
	4	30 (7.1)	
Sex (male), n (%)			330 (77.5)
Smoker, n (%)			52 (12.2)
Diabetes, n (%)			149 (35)
COPD^d^, n (%)			104 (24.4)
Recent diagnosis, n (%)			187 (43.9)
No β-blocker medication, n (%)			159 (37.3)
No ACE^e^ inhibitor medication, n (%)			79 (18.5)
Myocardial infarction, n (%)			242 (56.8)
Chronic atrial fibrillation, n (%)			112 (26.3)

^a^EF: ejection fraction.

^b^SBP: systolic blood pressure.

^c^NYHA: New York Heart Association.

^d^COPD: chronic obstructive pulmonary disease.

^e^ACE: angiotensin-converting enzyme.

### Base-Case Analysis

The base-case analysis was conducted in accordance with Dutch guidelines for economic evaluations in health care [30]. A societal perspective was adopted, which considered all costs inside the health care sector, patient and family sector, and other sectors, regardless of who is paying for those costs, as well as productivity losses assessed using the friction cost method [31], and future unrelated medical costs. All costs were reported in 2020 euros, where 2020 figures were not available, and older costs were inflated using the general price index from the Dutch Central Bureau of Statistics [32]. Health outcomes (effects) were presented in life years and QALYs and discounted at 4%, whereas costs were discounted at 1.5%. The analysis adopted a lifetime horizon, and the model was run for 1000 patients. An overview of the model input parameters is presented in Table 2 and is explained in detail in the following sections.

**Table 2:** Model input parameters.

Parameter (source)			Mean value	Probabilistic sensitivity analysis			Deterministic sensitivity analysis (95% CI)			Observations
				SE	Distribution					
**Model settings**										
	Discount rate (costs, %) [30]		4	N/A^a^	N/A	0-8				Dutch EE^b^ guidelines
	Discount rate (effects, %) [30]		1.5	N/A	N/A	0-3				Dutch EE guideline
	Time horizon [30]		Lifetime	N/A	N/A	N/A				Dutch EE guidelines
**Treatment effect**										
	Time-to-death (distribution) [29]		Weibull	N/A	N/A	N/A				Uncertainty assessed in the scenario analyses
	Time-to-hospitalization (distribution) [29]		Log-normal	N/A	N/A	N/A				Uncertainty assessed in the scenario analyses
	Time-to-outpatient visit (UC^c^, months) [17]		2.81	10% of the mean	Normal	2.46-3.13				None
	Time-to-outpatient visit (HTM^d^, months) [17]		1.69	10% of the mean	Normal	1.59-1.79				None
**Diagnostic algorithm**										
	Sensitivity [18]		0.52	N/A	N/A	N/A				Uncertainty assessed in the scenario analyses for the DA^e^
	False-positive rate [18]		0.03	N/A	N/A	N/A				Uncertainty assessed in the scenario analyses for the DA
	Proportion avoidable hospitalizations (%) [33]		50	20% of the mean	Normal	33.6-66.4				None
**Costs (€)**										
	Outpatient visit (UC) [17,19]		44.50	20% of the mean	Gamma	30.94-60.08				None
	Outpatient visit (HTM) [17,19]		43.30	20% of the mean	Gamma	30.11-58.46				None
	Other HF^f^-related care provider contacts (UC) [17,19]		188.38	20% of the mean	Gamma	130.98-254.33				None
	Other HF-related care provider contacts (HTM) [17,19]		623.61	20% of the mean	Gamma	433.59-841.93				None
	Hospitalization [17,19,20]		4404.46	20% of the mean	Gamma	3062.36-5946.44				None
	HTM device (per year) [21]		1257.75	20% of the mean	Gamma	1059.87-1469.69				None
	Managing alarm [19]		18.38	20% of the mean	Gamma	12.78-24.81				None
	Drug costs (per year) [17,22]		286.44	20% of the mean	Gamma	199.16-386.72				None
	Traveling expenses (outpatient visit) [17,19,23]		3.75	20% of the mean	Gamma	2.61-5.06				None
	Traveling expenses (hospitalization) [19,23]		4.68	20% of the mean	Gamma	3.25-6.32				None
	Informal care (per year) [17,19,24,34]		2098.28	20% of the mean	Gamma	1458.90-2832.88				None
**Utilities**										
	NYHA^g^ class I [21]		0.87976	0.00827	Beta	0.86588-0.89308				None
	NYHA class II [21]		0.71178	0.00944	Beta	0.69615-0.72720				None
	NYHA class III [21]		0.61405	0.01349	Beta	0.59176-0.63614				None
	NYHA class IV [21]		0.49228	0.03032	Beta	0.44243-0.54220				None
	Utility multiplier (outpatient visit)		1	N/A	N/A	N/A				Assumption; excluded from uncertainty analyses^h^
	Utility multiplier (hospitalization) [35]		0.82	10% of the mean	Normal	0.69-0.95				None

^a^N/A: not applicable.

^b^EE: economic evaluation.

^c^UC: usual care.

^d^HTM: home telemonitoring.

^e^DA: diagnostic algorithm.

^f^HF: heart failure.

^g^NYHA: New York Heart Association.

^h^Depending on the rate of outpatient visits, positive values may generate higher quality-adjusted life years when compared with life years.

### Treatment Effect of HTM (Compared With Usual Care)

When compared with usual care, HTM is modeled to increase time-to-hospitalization and time-to-death while decreasing time-to-outpatient visits.

The treatment effect of HTM on time-to-hospitalization and time-to-death was modeled using parametric models (exponential, Weibull, log-normal, log-logistic, Gompertz, and generalized gamma) fitted to empirical time-to-hospitalization and time-to-death data (Kaplan-Meier curves) for HTM and usual care from the TEN-HMS trial [17]. The models assumed proportional hazards between HTM and usual care. In the base-case analysis, a Weibull distribution was used to extrapolate time-to-death and a log-normal distribution to extrapolate time-to-hospitalization. The distributions were chosen according to the recommendations issued by the Decision Support Unit commissioned by the National Institute for Health and Clinical Excellence [36]. Details of the survival analysis can be found in the original publication of the model [29].

To predict in-hospital patient mortality, we ran a logistic regression where the probability of dying in the hospital was explained by age, sex, previous history of myocardial infarction or chronic atrial fibrillation, comorbidities (diabetes or chronic obstructive pulmonary disease), and the number of previous hospitalizations.

The time-to-outpatient visit was a parameter set by the user in the model. There is no periodic outpatient visit suggested in Dutch or international guidelines, as it is recommended that the time to the next consultation be scheduled by the accompanying physician and based on the clinical status of the patient [37,38]. Therefore, we assumed that the time-to-outpatient visit for the population under analysis is properly represented by the observations in the TEN-HMS study [17]: 2.81 months for usual care and 1.69 months for HTM-based interventions. This assumption is strengthened by the fact that 37.8% (161/426) of patients included in the TEN-HMS trial were treated in Dutch hospitals [17].

### Treatment Effect of the DA (When Added to HTM)

To model the treatment effect of adding the DA to the HTM, we considered the algorithm as a binary test for predicting hospitalization. Depending on the threshold value for the alarm of the DA, it has a certain sensitivity and specificity. The treatment effect of the DA is included in the model through its sensitivity and false-positive rate (same as 1 − specificity).

Sensitivity corresponds to the probability of correctly predicting a hospitalization when that would be the next event to be processed in the model. Hospitalization is avoided in the simulation when it is correctly detected and clinically avoidable; the latter is approximated by the average for potentially preventable hospitalizations in heart failure reported in the literature, which is 50% [33]. Thus, assuming the sensitivity of the alarm is 0.52 and that 50% of hospitalizations are clinically avoidable, 0.52×50%=26% would be the overall probability of avoiding hospitalization.

The false-positive rate represents the proportion of false-positive alarms. Hence, if the false-positive rate of the DA (with daily alarms) was 0.03 and there were 100 days between the previous and current events simulated in the model, there would be 3 false-positive alarms during the period between both events. The false-positive alarms are included in the model through the cost of managing those alarms, and they are assumed to have no consequences for health outcomes.

In our study, we used the DA developed using multiresolution analysis signals for diastolic blood pressure and weight collected daily by a noninvasive HTM for predicting hospitalization published elsewhere [18]. The sensitivity and false-positive rate in the base-case analysis were set to the figures reported in that study: 0.52 and 0.03, respectively.

### Outpatient Visit Costs

The office visits reported in the TEN-HMS trial discriminated among general practitioner, nurse, and specialist visits for both usual care and HTM [17]. We assumed that this partition was representative of Dutch clinical practices for the population under analysis. Through calculating the weighted average between the product of the visit type and its reference price in the Dutch Costing Manual [19], we estimated the costs of an outpatient visit to be €44.50 for usual care and €43.30 for HTM (Table S1 in Multimedia Appendix 1).

### Costs of Other Heart Failure–Related Care Provider Contacts

The number and type of health care resources used (emergency room visits, office visits, home visits, and telephone calls) during the TEN-HMS trial were reported for usual care and HTM for 240 follow-up days [17]. The TEN-HMS data were also assumed to represent Dutch clinical practices for heart failure management. To estimate the costs of other heart failure–related care provider contacts, we excluded office visits, as they were used separately for estimating the cost per outpatient visit (see *Outpatient Visit Costs* section). We converted the resources used during the follow-up period in the TEN-HMS trial (240 days) to yearly rates per patient and multiplied these figures by the cost of the resources included in the Dutch Costing Manual [19]. The estimated costs of contact with other heart failure–related care providers per year were €188.38 for usual care and €623.61 for HTM (Table S2 in Multimedia Appendix 1).

### Hospitalization Costs

The average hospital stay in the Netherlands for heart failure was 8.6 days for men and 8.4 days for women [20]. The sex partition of the population included in the TEN-HMS trial was 77.5% (330/426) men and 22.5% (96/426) women [17]. Using the average cost of a hospital day from the Dutch Costing Manual [19] and the weighted average of hospital days according to sex, we estimated the average costs per hospitalization at €4404.46 (Table S3 in Multimedia Appendix 1).

### HTM Costs

We used the midpoint of the telemonitoring costs from the range of yearly equipment and service fees and the installment fee (every 5 years) reported elsewhere [21] to obtain a yearly cost estimate of €1257.75 for HTM. In addition, we used the cost for a general practitioner teleconsultation reported in the Dutch Costing Manual [19] (€18.38) to manage false-positive alarms raised by the DA (Table S4 in Multimedia Appendix 1).

### Drug Costs

The TEN-HMS database contains information about the drugs used by each patient. Every drug reported to have been used in >5% of the total patients was included in the cost analysis. The daily dose assumptions for each drug were obtained from figures reported elsewhere [25] and confirmed by expert opinion. The representativeness of the TEN-HMS trial for Dutch clinical practices for the considered population is discussed earlier in the text and assumed for drug use.

The daily drug costs were based on the cheapest option available in the Z-index [22] and calculated using the following formula from the Dutch Costing Manual [19]: Drug costs=pharmacists purchase price (Z-index)−clawback (8.3%)+value added tax (6%)+pharmacy dispensing fee. The pharmacy dispensing fee was calculated by dividing the total fee by the number of units in the considered presentation and multiplying it by the number of units taken daily. The costs of insulin therapy were not available in the Z-index database and were extracted from the literature [26].

The total average drug cost per patient per year was estimated at €286.44 (Table S5 in Multimedia Appendix 1 shows the breakdown of drug costs included in the model).

### Informal Care Costs

The TEN-HMS database contained information on the burden to others reported at baseline for 98.6% (420/426) of the patients. Possible answers were *no*, *very little*, *a little*, *some*, *a lot*, and *very much*. These were modeled to correspond to 0%, 2%, 4%, 6%, 8%, and 10% of the time spent on informal care during a 16-hour day, respectively. After analyzing these data based on the New York Heart Association (NYHA) classification, we determined that there were no significant differences between classes (Table S6 in Multimedia Appendix 1), and we used the average of the whole population to obtain informal care costs. The total average cost of informal care per patient per year (€2098.28) was obtained by multiplying the average hours of informal care per 16-hour day by 365.25 days and by the hourly cost of informal care from the Dutch Costing Manual [19] (Table S7 in Multimedia Appendix 1).

### Traveling Expenses

Traveling expenses were calculated based on Kanters et al [23] and added to the costs of outpatient visits and hospitalizations (Table S8 in Multimedia Appendix 1).

### Costs Related to Productivity Losses

Because we used a patient-level simulation model, we could include age- and sex-specific productivity costs for each individual patient until 65 years of age, after which we assumed that patients did not incur further productivity costs.

Productivity losses were assigned to hospitalizations of patients who were considered working at baseline. We assumed that a hospitalized patient incurs productivity costs for 1 whole month, as it seems unlikely that the patient will be able to return to work immediately after being hospitalized. We further assumed that the working status did not change during the model, which led to the exclusion of long-term productivity costs from the model. We used the proportion of patients assumed to be working per NYHA class based on expert opinions reported elsewhere [25]. The working probability of each patient was adjusted using an age- and sex-specific net labor participation rate for the general population [34]. The total cost per day was calculated using age- and sex-specific data on working hours per week and hourly labor cost [24] (Table S9 in Multimedia Appendix 1 shows the inputs for the calculation of productivity costs, and Table S10 in Multimedia Appendix 1 shows an example of the costs incurred by a hypothetical patient).

### Future Unrelated Medical Costs

Dutch guidelines require the inclusion of additional costs from unrelated diseases during the life years gained with interventions that extend life expectancy [30]. We extracted the estimates of per capita health care expenditures based on age and sex from the Practical Application to Include Disease Costs 3.0 tool and included those costs for each patient individually during the simulation [27,39] (Table S11 in Multimedia Appendix 1).

### Health Outcomes and Utilities

QALYs were obtained by weighing life years with patient utility over time. Utilities were attributed to each patient at the start of the simulation according to their NYHA class at baseline and to NYHA class-specific utility values reported elsewhere [21] (Table S12 in Multimedia Appendix 1). The utilities change over time with events occurring in the simulation. It was assumed that there were no utility changes resulting from outpatient visits and that hospitalizations resulted in a decrease in utility by a factor of 0.82, following the change in utility observed between NYHA classes reported in another study published for a similar heart failure population [35]. We assumed that the disutility factor from hospitalization should be limited to 3 events.

On the basis of the equation estimated by Ara and Brazier for the utilities for the general UK population (equation S1 in Multimedia Appendix 1), age-sex-specific utilities attributed at baseline were capped, and a decrement factor for aging was implemented [28].

### Cost-effectiveness

The average outcome per patient is presented for each intervention. The incremental cost-effectiveness ratio (ICER) was calculated as the difference in the average total cost per patient divided by the difference in the average number of QALYs per patient (€/QALY). The calculated ICER was then compared with the Dutch cost-effectiveness threshold. The intervention can be considered cost-effective if the calculated ICER is lower than the appropriate cost-effectiveness threshold for the population and the situation under analysis.

The cost-effectiveness threshold in the Netherlands depends on the burden of disease as measured by the fraction of QALYs that people lose relative to the situation in which the disease had been absent (proportional shortfall) [40-42]. The appropriate cost-effectiveness threshold, which represents the societal willingness to pay for an additional QALY for that specific patient population, can be calculated using the Institute for Medical Technology Assessment Disease Burden Calculator [43].

### Sensitivity and Scenario Analyses

Parameter uncertainty was assessed using deterministic sensitivity analyses [44]. The joint parameter uncertainty was explored through probabilistic sensitivity analysis, including the parameter distributions specified in Table 2 [45,46]. Following the methodology for addressing uncertainty in discrete event simulation models published elsewhere [47], probabilistic sensitivity analysis was implemented as a double loop: an inner loop, in which a predetermined number of patients were sampled with replacement from the baseline population, and an outer loop, in which the values of the input parameters of the model were randomly drawn. The results of a probabilistic sensitivity analysis with an inner loop of 100 patients and an outer loop of 500 iterations were plotted on the cost-effectiveness plane [46,48,49]. Cost-effectiveness acceptability curves were drawn [50,51].

Scenario analyses were run, in which key structural assumptions regarding time-to-death and time-to-hospitalization parametric survival models, time-to-outpatient visits, utilities, and costs were varied to estimate the impact of these assumptions on the outcomes.

### Value of Information Analysis

The guidelines for economic evaluations in the Netherlands require calculation of the expected value of perfect information (EVPI) when the probability that the intervention is cost-effective at the appropriate cost-effectiveness threshold is <100% [30]. EVPI per patient is calculated as the average of the maximum net benefits in each probabilistic sensitivity analysis iteration minus the maximum average net benefit for the interventions considered in the analysis [52-54]. The population EVPI is calculated by multiplying EVPI per patient by the size of the potential population benefiting from the new intervention across the time span for which the recommendation resulting from the value of information analysis is applicable. We assumed 5 years for the expected applicability of the recommendation, and we estimated the number of patients eligible for HTM-based interventions in the Netherlands from 2020 to 2024 to be 53,140, 55,009, 56,943, 58,946, and 61,019 [55-57]. We discounted EVPI at 4% per year.

### Cost-effectiveness of the DA

In the context of the predictive performance of binary diagnostic tests, an ROC curve is a graph that illustrates the diagnostic ability of a binary classifier system by plotting the sensitivity values against the false-positive rates (1−specificity) at various threshold settings.

To properly assess the cost-effectiveness of the DA when added to the HTM intervention, we ran the model at different points of the ROC curve of the DA other than the base-case scenario, thus inferring at which combinations of sensitivity and specificity the DA would be the most cost-effective. In other words, this analysis aimed to determine the operating point at which the threshold of the DA should be set to achieve the best balance between costs and health outcomes for the HTM+DA intervention. The values of sensitivity and false-positive rate were measured using Graphreader [58].

### Subgroup Analyses

We analyzed a wide range of subgroups by varying patient and disease characteristics, as presented in Table 1. We created 2 subgroups based on age (<65 and ≥65 years) and 2 subgroups based on the ejection fraction (<25% and >25%). We further analyzed patients belonging to each NYHA class separately, creating 4 subgroups. Finally, each dichotomous variable generated 2 subgroups (characteristic present or not present). In total, we analyzed 26 patient subgroups.

### Ethics Approval

As this is a mathematical simulation study, ethics approval was not applicable.

## Results

### Base-Case Analysis

The main results of the base-case analysis are summarized in Table 3 (average outcomes per patient) and Table 4 (incremental cost-effectiveness ratios).

Usual care patients experienced approximately 3 outpatient visits per year less than HTM-based interventions. Conversely, HTM results in a decrease in the yearly rate of hospitalizations compared with usual care (1.64 vs 1.70). This decrease is even more pronounced when the DA is added to HTM, as 0.45 (95% CI 0-2.12) hospitalizations per year are avoided owing to the DA.

Usual care was the intervention with the lowest total discounted costs (€46,879), followed by HTM (€60,343), and HTM+DA (€65,008). On average, patients were expected to survive 2.18 discounted years with usual care, 2.96 with HTM, and 3.44 with HTM+DA, corresponding to 1.12, 1.51, and 1.78 discounted QALYs, respectively. The hierarchical analysis of the costs and QALYs of the 3 interventions showed that HTM is extendedly dominated by HTM+DA, as the ICER of HTM compared with usual care (€34,449/QALY) is higher than that of HTM+DA (the next, more effective, alternative) compared with usual care (€27,712/QALY).

The standardized quality-adjusted life expectancy for the population included in the analysis (approximately 67 years of age and 78% of male patients) was 14.7 QALYs. The total expected undiscounted QALYs accrued with the current standard of care (usual care) in the model being 1.16, which indicates that 92.1% of normal quality-adjusted life expectancy is lost owing to the disease. In this situation, the appropriate cost-effectiveness threshold using the proportional shortfall approach was €80,000 per QALY.

**Table 3:** Average outcomes per patient in the base-case analysis (n=1000).

Average outcomes per patient		UC^a^	HTM^b^	HTM+DA^c^
**Intermediate outcomes (events per year)**				
	Outpatient visits	3.60	6.62	6.63
	Hospitalizations	1.70	1.64	1.31
	Avoided hospitalizations	—^d^	—	0.45^e^
**Death type**				
	Death in hospital, n (%)	472 (47.2)	642 (64.2)	585 (58.5)
	Death (other), n (%)	528 (52.8)	358 (35.8)	415 (41.5)
**Final outcomes (discounted)**				
	Total costs (€)	46,879	60,343	65,008
	Total life years	2.18	2.96	3.44
	Total QALYs^f^	1.12	1.51	1.78

^a^UC: usual care.

^b^HTM: home telemonitoring.

^c^DA: diagnostic algorithm.

^d^Not available.

^e^Avoided hospitalizations within the HTM+DA intervention group.

^f^QALY: quality-adjusted life year.

**Table 4:** Incremental cost-effectiveness ratios.

Incremental cost-effectiveness analysis	HTM^a^ vs UC^b^	HTM+DA^c^ vs HTM^d^	HTM+DA vs UC
Δ€	13,465	4665	18,129
ΔQALY^e^	0.39	0.26	0.65
Δ€/ΔQALY	€34,449^f^	€17,713	€27,712

^a^HTM: home telemonitoring.

^b^UC: usual care.

^c^DA: diagnostic algorithm.

^d^Extendedly dominated by HTM+DA. Extended dominance was investigated by ranking the 3 interventions (HTM+DA, HTM, and UC) according to their effectiveness and calculating the ICER to the next best alternative (ie, HTM+DA vs HTM and HTM vs UC). When the cost-effectiveness of HTM versus UC is worse, that is, the ICER is higher than that of HTM+DA vs HTM, HTM is extendedly dominated by HTM+DA. HTM should not be adopted because a combination of the standard of care (UC) and the most effective treatment alternative (HTM+DA) generates better outcomes than the extendedly dominated treatment alternative (HTM).

^e^QALY: quality-adjusted life year.

### Sensitivity and Scenario Analyses

**Figure 1:**
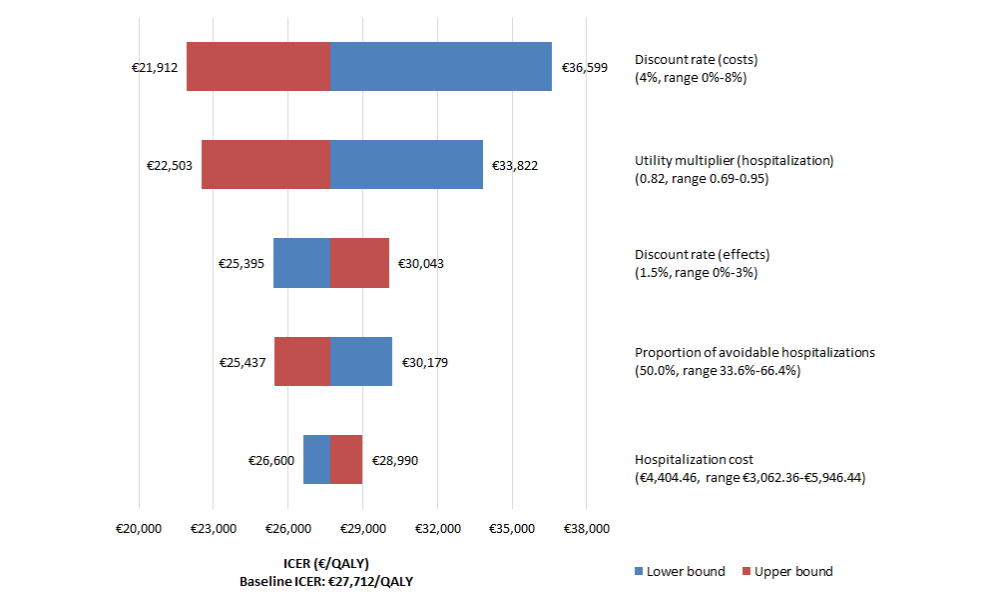
Tornado diagram for the home telemonitoring plus diagnostic algorithms vs usual care comparison. ICER: incremental cost-effectiveness ratio; QALY: quality-adjusted life year.

Considering the extended dominance of HTM+DA over HTM, univariate sensitivity analyses were performed only for the HTM+DA versus usual care comparison. The results of the 5 input parameters with the largest effects on the ICER are presented in the tornado diagram in Figure 1. All ICERs remained below the threshold of €80,000/QALY.

**Figure 2:**
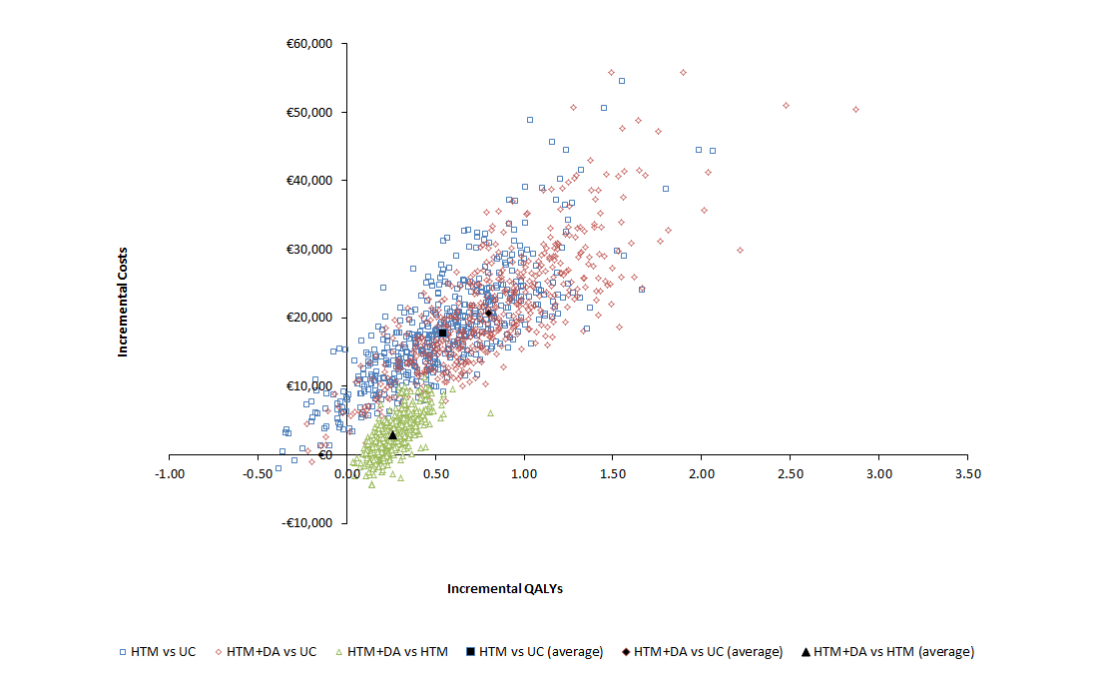
Incremental cost-effectiveness plane. DA: diagnostic algorithm; HTM: home telemonitoring; QALY: quality-adjusted life year; UC: usual care.

**Figure 3:**
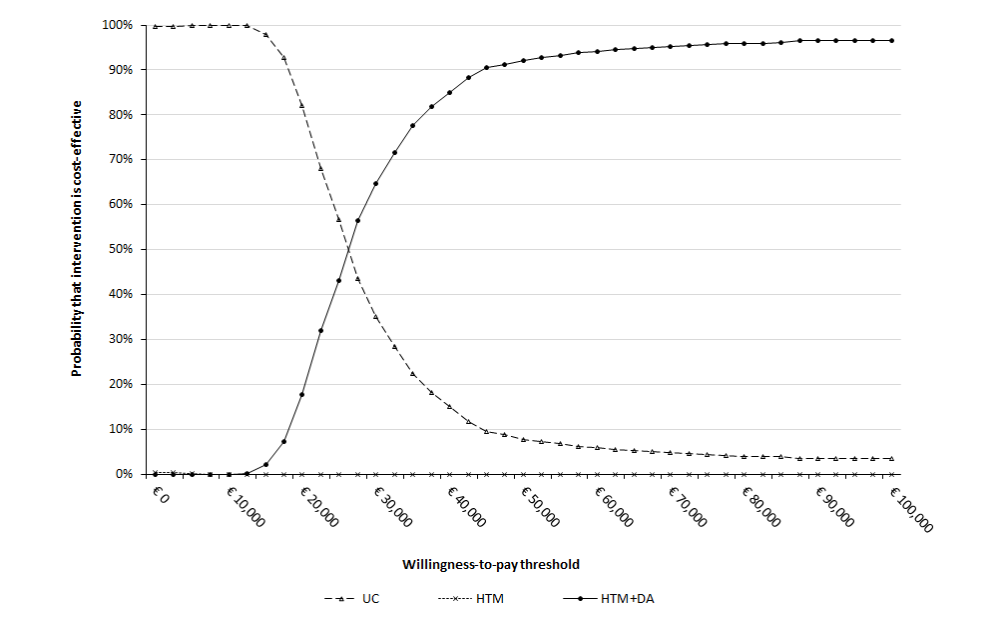
Cost-effectiveness acceptability curve. DA: diagnostic algorithm; HTM: home telemonitoring; UC: usual care.

The probabilistic sensitivity analysis outcomes plotted in the cost-effectiveness plane for each pairwise comparison show that the great majority of simulations fall in the northeast quadrant; that is, interventions have higher costs and accrue more QALYs than their comparators (Figure 2). The probabilistic ICER between HTM+DA and usual care was similar to that found in the base-case analysis: €25,864/QALY (95% CI 15,527-54,151). The cost-effectiveness acceptability curves for the 3 interventions show that usual care is expected to be the most cost-effective at low willingness-to-pay thresholds, HTM is never the most cost-effective intervention, and HTM+DA becomes the intervention most likely to be cost-effective from €25,864 per QALY upward, reaching a 96.0% probability at the appropriate cost-effectiveness threshold of €80,000 per QALY (Figure 3).

The results of the scenario analyses assessing the structural assumptions of the model are summarized in Table S13 in Multimedia Appendix 1. The scenario with the highest impact on the ICER was the one where a health care perspective was taken, which resulted in an ICER between HTM+DA and usual care of €14,408/QALY (−48.0% when compared with the base-case analysis). In contrast, the scenario taking all costs from the upper bound of the 95% CIs was the one with the highest ICER (€31,829/QALY). All ICERs from the scenario analyses remained below the threshold of €80,000 per QALY.

### Value of Information Analysis

In the base-case analysis, at the appropriate threshold of €80,000 per QALY, the probability of HTM+DA being cost-effective was 96.0%. The calculated EVPI per patient was €341. With an estimated number of patients eligible for the HTM-based interventions in the Netherlands being 253,118 (after discounting) from 2020 to 2024, the population EVPI was estimated at €86,383,575.

### Cost-effectiveness of the DA

The results for the treatment scenarios, assuming different characteristics of the DA, are presented for the comparison of HTM+DA with usual care in Table 5. Increasing the sensitivity of the DA by setting a lower threshold for the alarm to go off, which entails an increase in the false-positive rate (decreased specificity), resulted in a higher number of avoided hospitalizations, life years, and QALYs, but with higher costs. Alternatively, decreasing sensitivity (ie, setting a higher threshold for the alarm) resulted in lower costs but worse health outcomes. From the scenarios tested, the most cost-effective was scenario 3, where the sensitivity was set to 0.600 and the false-positive rate to 0.068. In the scenario tests, moving away from that point in either direction of the ROC curve resulted in higher ICERs (ICER range: €25,734/QALY-€35,560/QALY).

**Table 5:** Results of the scenario analyses for the diagnostic algorithm (DA).

Average outcomes per patient (HTM^a^+DA)			DA scenarios										
			1 (sens^b^: 0.200; FPR^c^: 0.007)		2 (sens: 0.400; FPR: 0.024)		BC^d^ (sens: 0.520; FPR: 0.030)		3 (sens: 0.600 FPR: 0.068)		4 (sens: 0.800; FPR: 0.194)		5 (sens: 0.950; FPR: 0.562)
**Intermediate outcomes (events per year)**													
	Outpatient visits	6.63		6.64		6.63		6.62		6.62		6.63	
	Hospitalizations	1.52		1.36		1.31		1.23		1.10		1.00	
	Avoided hospitalizations	0.18		0.33		0.45		0.56		0.76		0.92	
**Final outcomes**													
	Total costs, €	62,085		63,394		65,008		64,163		71,016		82,108	
	Total life years	3.14		3.29		3.44		3.43		3.73		3.99	
	Total QALYs^e^	1.61		1.70		1.78		1.80		1.96		2.11	
**ICER^f^**													
	Versus UC^g^ (€/QALY)	30,984		28,881		27,712		25,734		29,004		35,560	
	Change vs base case (%)	+11.8		+4.2		0		−7.1		+4.7		+28.3	

^a^HTM: home telemonitoring.

^b^sens: sensitivity.

^c^FPR: false-positive rate.

^d^BC: base case.

^e^QALY: quality-adjusted life year.

^f^ICER: incremental cost-effectiveness ratio.

^g^UC: usual care.

### Subgroup Analyses

A summary of the cost-effectiveness results of subgroup analyses is presented in Table 6. Because each subgroup was created from a subset of the population in the TEN-HMS database [17], the characteristics of the baseline population for each subgroup may differ. The baseline patient and disease characteristics of the model population for each of the analyzed subgroups are presented in Tables S1-S26 in Multimedia Appendix 2. All ICER changes versus the base-case concern the comparison between HTM+DA and usual care.

Although many other subgroups did not show such a high variation in the ICER, as this is a ratio that depends on the simultaneous variation of costs and QALYs for each of the interventions being compared, large differences in the final outcomes were observed for some subgroups. Male patients (especially when compared with female patients) and patients from NYHA class III, with diabetes, with chronic obstructive pulmonary disease, not on β-blocker medication, not on angiotensin-converting enzyme medication, with a history of myocardial infarction, and with a history of chronic atrial fibrillation showed a considerable decrease in QALYs for both HTM+DA and usual care. For those subgroups, given that we were dealing with dichotomous variables, the complementary subgroups resulted in higher QALYs (ie, better health outcomes), with the exception of smokers versus nonsmokers, where the comparison showed small differences in QALYs and costs.

For all subgroups that showed a decrease in QALYs, a decrease in costs was also observed. This corroborates the positive correlation between costs and effects that were noticeable in the incremental cost-effectiveness plane shown in Figure 2. Hence, a decrease in life expectancy, and therefore QALYs, is associated with increased ICERs when compared with the base-case analysis.

**Table 6:** Subgroup analyses: summary of cost-effectiveness results.

Number	Subgroup^a^	Costs (€)			QALYs^b^				ICER^c^ (€/QALY)	
		UC^d^	HTM^e^	HTM+DA^f^	UC	HTM^g^	HTM+DA	HTM+DA vs UC		Percentage vs base case
—	Baseline population	46,879	60,343	65,008	1.12	1.51	1.78	27,712		0.0
1	Age <65 years	59,543	75,311	79,144	1.78	2.25	2.64	22,830		−17.6
2	Age ≥65 years	39,380	52,035	56,483	0.82	1.14	1.32	34,368		+24.0
3	Ejection fraction <25%	45,516	60,745	64,906	1.22	1.67	1.94	26,813		−3.2
4	Ejection fraction ≥25%	46,843	61,279	65,606	1.06	1.39	1.65	31,372		+13.2
5	NYHA^h^ class I^i^	53,679	72,656	77,377	1.84	2.51	2.88	22,870		−17.5
6	NYHA class II	48,659	64,094	67,515	1.24	1.63	1.90	28,827		+4.0
7	NYHA class III	43,142	51,046	54,454	0.80	1.00	1.18	29,759		+7.4
8	NYHA class IV	36,821	45,218	48,957	0.38	0.50	0.61	52,727		+90.3
9	Sex: male	45,762	57,518	61,122	1.08	1.36	1.60	29,777		+7.5
10	Sex: female	51,148	68,937	75,954	1.53	2.05	2.38	29,038		+4.8
11	Smoker: yes	49,819	62,956	64,973	1.18	1.48	1.73	27,765		+0.2
12	Smoker: no	45,741	60,614	64,392	1.13	1.49	1.74	30,208		+9.0
13	Diabetes: yes	43,213	55,144	59,211	0.96	1.26	1.48	30,624		+10.5
14	Diabetes: no	48,611	60,287	65,193	1.27	1.59	1.86	27,980		+1.0
15	COPD^j^: yes	39,386	47,599	52,293	0.80	1.02	1.24	29,560		+6.7
16	COPD: no	49,180	67,014	70,128	1.23	1.67	1.92	30,105		+8.6
17	Recent diagnosis: yes	54,103	69,207	74,122	1.53	1.90	2.20	29,748		+7.3
18	Recent diagnosis: no	42,619	53,123	56,272	0.91	1.18	1.39	28,567		+3.1
19	No β-blocker medication: yes	38,967	48,709	50,661	0.70	0.92	1.09	29,830		+7.6
20	No β-blocker medication: no	51,211	67,252	71,213	1.41	1.85	2.15	27,127		−2.1
21	No ACE^k^ inhibitor medication: yes	39,967	52,165	54,888	0.76	1.04	1.21	32,921		+18.8
22	No ACE inhibitor medication: no	47,208	61,294	65,897	1.20	1.57	1.84	29,424		+6.2
23	Myocardial infarction: yes	43,366	57,360	61,261	0.99	1.33	1.56	30,958		+11.7
24	Myocardial infarction: no	51,222	64,252	69,338	1.41	1.79	2.10	26,195		−5.5
25	Chronic atrial fibrillation: yes	38,205	49,469	53,452	0.72	1.02	1.19	32,415		+17.0
26	Chronic atrial fibrillation: no	50,164	64,086	68,856	1.31	1.71	2.01	26,812		−3.2

^a^Because each subgroup was created from a subset of the population in the TEN-HMS database [17], the characteristics of the baseline population for each subgroup may differ. The baseline patient and disease characteristics of the model population for each of the analyzed subgroups are presented in Tables S1-S26 in Multimedia Appendix 2.

^b^QALY: quality-adjusted life year.

^c^ICER: incremental cost-effectiveness ratio.

^d^UC: usual care.

^e^HTM: home telemonitoring.

^f^DA: diagnostic algorithm.

^g^HTM is extendedly dominated by HTM+DA in all analyzed subgroups. The ICER comparison against the base case is shown only for HTM+DA versus UC.

^h^NYHA: New York Heart Association.

^i^The subgroup of patients with NYHA class IV registered the highest deviation from the base-case analysis results, with an ICER of €52,727/QALY (+90.3%). By contrast, the subgroups with better cost-effectiveness ratios were patients <65 years of age and patients belonging to NYHA class I (22,830/QALY [−17.6%] and €22,870/QALY [−17.5%], respectively).

^j^COPD: chronic obstructive pulmonary disease.

^k^ACE: angiotensin-converting enzyme.

## Discussion

### Principal Findings

This study aimed to assess the cost-effectiveness of HTM and a DA in the management of heart failure in the Netherlands. It used a previously validated patient-level discrete event simulation model [29] for analyzing 3 separate interventions: usual care, HTM, and HTM+DA. The base-case analysis determined that HTM is extendedly dominated by HTM+DA, with the latter intervention being cost-effective versus usual care at a deterministic ICER of €27,712 per QALY gained (Table 4).

The cost-effectiveness of the DA was carefully examined through creating various scenarios with different values for sensitivity and false-positive rate from the ROC curve published by Koulaouzidis et al [18]. These scenarios generated model outcomes that allowed for the comparison of the ICER of HTM+DA versus usual care at various thresholds of the DA (Table 5), thereby assessing the inherent trade-off between false positives and false negatives in cost-effectiveness terms. In this study, false positives corresponded to alarms that were incorrectly raised, as the patient would not have been hospitalized, whereas false negatives represented alarms that were correctly raised and thus did not possibly avoid hospitalization. In the DA scenarios tested, scenario 5 minimized false negatives at the expense of increasing the number of false positives. Conversely, scenario 1 minimizes false positives at the expense of increasing false negatives. Although both false positives and false negatives are undesirable, there is an optimal point in terms of cost-effectiveness, which represents the balance between sensitivity and false-positive rate within the ROC curve in terms of generated QALYs and associated costs. In our analysis, scenario 3 is closer to this optimal point, as it leads to the lowest ICER of HTM+DA compared with usual care.

Subgroup analyses showed considerable variation in the ICERs of HTM+DA versus usual care (Table 6), with the highest ratios recorded for the subgroups of patients ≥65 years of age and those in NYHA class IV. A large variation in costs and QALYs was also observed, even when the resulting ICER did not change significantly from the base-case analysis for the HTM+DA versus usual care comparison, which may be attributed to the positive correlation between costs and effects observed in the subgroup analyses. It was also observed that complementary subgroups (with the exception of smokers or nonsmokers) went in opposite directions in relation to final outcomes (eg, lower QALYs and costs for patients with a history of myocardial infarction contrasted with higher QALYs and costs for patients without any history of myocardial infarction).

Deterministic sensitivity analyses and scenario analyses showed that the model results were robust to the variation of most parameters (Figure 1) and to most changes in structural assumptions, with the highest change in the ICER resulting from taking a health care perspective in the analysis. Probabilistic sensitivity analysis revealed a 96.0% chance of HTM with the addition of the DA being cost-effective at the appropriate threshold of €80,000/QALY (as determined by the proportional shortfall method).

### Practical Implication of Study Findings

From the point of view of clinicians, the findings of this study suggest an improvement in health outcomes when using the HTM system in the management of heart failure, especially when the DA is added. Thus, the results of this study support a change in the clinical practice for managing patients with heart failure, namely through the inclusion of the aforementioned health technologies.

The cost-effectiveness analysis presented in this paper relies on several distinguishing features of the Dutch economic evaluation guidelines: the adoption of a societal perspective, the calculation of productivity losses by using the friction cost method, differential discounting, the inclusion of caregiver burden on the cost side of the economic evaluation, the incorporation of indirect medical costs of life years gained, and the value of information analysis. Considering that the study followed all the methodological requirements for informing decision-making in the Netherlands, the financing of HTM and the DA in the Dutch health care system should be ensured.

Although this study only analyzed the cost-effectiveness of a particular HTM intervention and a DA, it serves to raise awareness that the arsenal for providing care is becoming more diverse and that the methodology for properly assessing new health technologies should follow that trend. The Federal Institute for Drugs and Medical Devices in Germany assesses digital health applications for reimbursement [59]. Other countries’ policy makers ought to learn from this experience and collaboratively work on solutions for the assessment of health care interventions supported by digital technologies, eHealth, and mHealth, particularly with regard to their cost-effectiveness. Only a correct assessment of their cost-effectiveness, which is a key criterion for deciding on the reimbursement of a new health technology in most developed health care systems, can result in an appropriate resource allocation within the present health care panorama.

### Comparison With Prior Work

To our knowledge, this is the first study to use a health economic patient-level simulation model to assess the cost-effectiveness of heart failure intervention in the Netherlands. Concerning the intervention, 2 studies have also assessed the cost-effectiveness of HTM in the Netherlands (Boyne et al [60] and Grustam et al [3,21]). Boyne et al [61,62] performed a trial-based economic evaluation of the Telemonitoring in Heart Failure (TEHAF) study, a prospective open-label, multicenter, randomized controlled trial with blinded endpoint evaluation, conducted at 3 hospitals in the Netherlands. The results of this study cannot be compared with those of our study. First, because the population in the TEHAF study was in a better health state than that in the TEN-HMS study (eg, mean ejection fraction of 36% vs 25%), and second, because the time horizon of their study was only 1 year, which cannot properly capture the lifetime change in costs and effects between the interventions because patients are expected to survive for >1 year. Grustam et al [21] used a Markov cohort model with most of the data from the TEN-HMS study to assess the cost-effectiveness of HTM compared with usual care. They took a third-party payer’s perspective, and a direct comparison of results with that study would be unwise and uninformative. However, in the scenario analysis where we took a health care perspective (scenario 23 in Table S13 in Multimedia Appendix 1), we estimated similar costs: €16,034 for usual care and €25,433 for HTM versus €14,414 and €27,186, respectively, as found by Grustam et al [21]. However, the ICERs were different because we estimated fewer QALYs. One possible explanation is the assumption by Grustam et al [21] that the transition probabilities measured in the time frame of 240 to 450 days in the TEN-HMS study continued unaltered for 20 years, which, given the mean age of 67 years of the patients included in the model and their very poor health state, seems unlikely. This assumption may have overestimated the survival in their study. Another possible explanation for the aforementioned difference is the potential underestimation of survival in our study owing to the regression equation for in-hospital mortality. The regression equation that calculates time-to-death predicts all-cause mortality. Thus, patients dying in hospitals may result in some type of double counting of mortality owing to the inherent imprecision of data-driven estimates. If the predictions were 100% accurate, the model would predict the time of death flawlessly, which never happens in practice. However, given the higher number of hospitalizations experienced by patients in the intervention arms owing to their increased survival, the cost-effectiveness estimates, if anything, are conservative.

The findings in our study of lower mortality and hospitalizations with HTM-based intervention when compared with usual care are consistent with the results previously published in 2 network meta-analyses [63,64]. Regarding costs, we found an increase in total costs with HTM when compared with usual care. In a review by Inglis et al [64], the authors identified 3 studies reporting costs for *HTM versus usual care*; one reported a decrease in costs and 2 reported increases in costs due both to the cost of the intervention and to increased medical management [64].

It is worth mentioning that the structure of the model used in our study allowed us to explore the impact of adding a DA to HTM intervention. This is a critical aspect of our study as it is the first to assess the cost-effectiveness of a DA in the context of chronic disease management. Although we have used this concept in the context of heart failure intervention, it can be adapted for other disease areas. This subject has been discussed in a publication on the validation of the model used in this study [29].

### Limitations

The first limitation stems from the TEN-HMS study dating from 2005, which resulted in a large enough period for medical practice to have changed, especially because we are discussing technologies that are developed at a fast pace. The experience that results from the continuous use of these technologies can ultimately have an impact on their effectiveness and cost-effectiveness. Also related to the TEN-HMS study, it should be noted that drug use patterns and their costs pertain to the standards existing at the time of the trial. Even if standards in terms of therapeutic classes are not necessarily different, the drugs used are older and are likely cheaper than the more recent alternatives (this impact was assessed via scenario analysis). In addition, some inputs used in the model, namely the proportion of avoidable hospitalizations and the utility decrement resulting from a hospitalization, were already older in age and were used due to the lack of more recent estimates. Finally, there could be some variation in health care systems between patients included in the TEN-HMS study (United Kingdom, the Netherlands, and Germany), which was not accounted for in the model.

The second limitation relates to the DA ROC curve used for the analysis. Because the ROC curve was not obtained using the same population or HTM system, we assumed that the different levels of diagnostic accuracy of the DA, that is, the different points of the ROC curve, would also be applicable to the population in our model. The population used in the study by Koulaouzidis et al [18] seemed to be in a better health state than that in the TEN-HMS study [17] (eg, ejection fraction of 36.6% vs 25.1%). Ideally, we would have a DA constructed with TEN-HMS data, as we would want to optimize the threshold of a DA that would have been designed with the same HTM system. Thus, we could use the data generated by this system to continuously improve predictions of hospitalization and, consequently, improve the cost-effectiveness of the HTM+DA intervention.

Concerning subgroup analyses, it is critical to emphasize that their interpretation is a sensitive matter, as every subgroup is created from the baseline population by restricting the variables of interest to values compatible with the subgroup being analyzed. Thus, subgroups are likely to have different patient and disease characteristics when compared with the model population used in the base-case analysis (Tables S1-S26 in Multimedia Appendix 2). For instance, NYHA class IV patients were also older, on average, than the baseline model population. Hence, the outcomes from the model and their variation from the base-case analysis in that situation are not only dependent on the impact of NYHA IV but also on all other patient and disease characteristics that change in the subgroup population when compared with the base-case population. Thus, the correct interpretation of subgroup analyses requires a link with the patient and disease characteristics than can be correlated with the particular characteristic changes in any given subgroup.

There is a dimension of patient preferences that has not been assessed in this study. Moving from a face-to-face type of care to a remote environment implies a change in the behavior of patients and their interaction with the health care system, which should be assessed more carefully.

Finally, strictly speaking, the results presented in this study concern only the specific HTM intervention used in the TEN-HMS study and the DA presented by Koulaouzidis et al [18] (see the *Interventions* section). Although some qualitative extrapolation to similar technologies could be made, the quantitative results presented in this study are specific to the data generated in the TEN-HMS study and the study by Koulaouzidis et al [17,18]. It should also be noted that the outcomes of HTM systems depend on patient use of the system. Therefore, the effectiveness in the real world could vary from the efficacy found in a controlled clinical environment. As such, generalization of the results of this study to other HTM systems and patient populations should be performed carefully and informedly.

### Future Directions

The model could include individual drug costs and optimize the medication used at each processed event. To achieve this, patient characteristics should be updated at these events to define the correct medication for each patient. In doing so, the model would also capture the drug costs more accurately.

Further research must be conducted to better describe the DAs and follow-up actions they entail in clinical practice and disease pathways. Although the discrete event simulation framework allowed for the assessment of the cost-effectiveness of the DA, the potential of these models opens enormous possibilities for designing a model with highly detailed disease pathways for clinicians and decision makers who are less familiar with decision modeling in the context of the economic evaluation of health technologies. However, the increased complexity of models comes at the expense of the need for patient-level data to build and validate the model. Theoretically, all patient pathways after an alarm can be included in a discrete event simulation framework. The question is whether there would be reliable data on the outcomes for each of the pathways that could be conceived for reacting to an alarm. As is widely described in the health economics literature, models should abide by the principle of parsimony; that is, they should be as simple as possible to accurately reflect the problem under analysis and allow for making an informed decision.

### Conclusions

Although increased costs of adopting HTM and a DA in the management of heart failure may seemingly be an additional strain on scarce health care resources, the results of this study demonstrate that, by increasing patient life expectancy by 1.28 years and reducing their hospitalization rate by 23% when compared with usual care, the use of this technology may be seen as an investment, as HTM+DA in its current form extendedly dominates HTM and generates an extra QALY for a €27,712 investment. At the appropriate cost-effectiveness threshold of €80,000/QALY resulting from the proportional shortfall methodology used in the Dutch economic evaluation guidelines, HTM+DA had a 96.0% probability of being cost-effective.

## Appendix

Multimedia Appendix 1 Additional tables and figures.

Multimedia Appendix 2 Baseline patient and disease characteristics of the model populations used in subgroup analyses.

## Abbreviations

DA: diagnostic algorithm
EVPI: expected value of perfect information
HTM: home telemonitoring
ICER: incremental cost-effectiveness ratio
NYHA: New York Heart Association
QALY: quality-adjusted life year
ROC: receiver operating characteristic
TEN-HMS: Trans-European Network—Home-Care Management System
