# Supplementary material for: Home Telemonitoring and a Diagnostic Algorithm in the Management of Heart Failure in the Netherlands: Cost-effectiveness Analysis
Source: JMIR Cardio. 2022 Aug 4;6(2):e31302. doi: 10.2196/31302 (PMC9389378; doi:10.2196/31302)
Supplement: Multimedia Appendix 1 [file cardio_v6i2e31302_app1.docx]

**Supplementary material**

(For the manuscript: Cost-effectiveness of a home telemonitoring system and a diagnostic algorithm in the management of heart failure in the Netherlands)


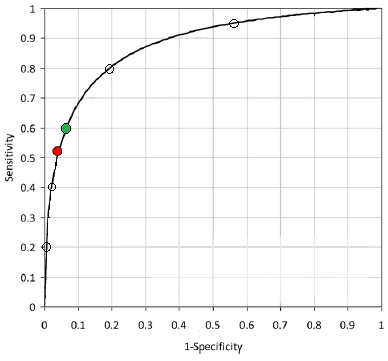


Figure S1 – Receiver operating characteristic curve used in the analysis. Adapted from Koulaouzidis et al. 2016 [18].

The red dot is the operating point for the base case analysis, the green dot is the operating point at optimal cost-effectiveness, and black circles are the remaining operating points used in the analysis.

Table S1 – Costs for outpatient visits for UC and HTM

|  |  | **UC** | | | **HTM** | | |
| --- | --- | --- | --- | --- | --- | --- | --- |
|  | Unit price [19] | Count [17] | % | Weighted cost (per visit) | Count [17] | % | Weighted cost (per visit) |
| GP | 35.69€ | 119 | 62.96% | 22.47€ | 454 | 69.42% | 24.77€ |
| Specialist | 98.41€ | 34 | 17.99% | 17.70€ | 100 | 15.29% | 15.05€ |
| Nurse | 22.71€ | 36 | 19.05% | 4.33€ | 100 | 15.29% | 3.47€ |
| ***Total*** |  | **189** | **100.00%** | **44.50€** | **654** | **100.00%** | **43.30€** |

Table S2 – Resource use and maintenance treatment cost calculations for UC and HTM

|  |  | **TEN-HMS count [17]** | |  | **Yearly rate per patient** | |  | **Reference prices [19]** | |  | **Maintenance treatment cost per patient per year** | | |
| --- | --- | --- | --- | --- | --- | --- | --- | --- | --- | --- | --- | --- | --- |
|  |  | **UC** | **HTM** |  | **UC** | **HTM** |  | **Resource** | **Unit price (2020€)** |  | **UC** | **HTM** | |
| **Follow-up days** |  | **240** | |  |  | |  |  |  |  |  | |  |
| ***Number of patients*** |  | 85 | 163 |  |  |  |  |  |  |  |  |  | |
| ***Total days at risk*** |  | 16089 | 33641 |  |  |  |  |  |  |  |  |  | |
| ***Emergency room visits*** |  |  |  |  |  |  |  |  |  |  |  |  | |
| *Visits* |  | 8 | 60 |  | 0.182 | 0.651 |  | *Reference emergency* | 280.10€ |  | 50.87€ | 182.47€ | |
| ***Office visits*** |  |  |  |  |  |  |  |  |  |  |  |  | |
| *Family practitioner* |  | 119 | 454 |  | 2.702 | 4.929 |  | *GP, standard consultation* | 35.69€ |  | 96.41€ | 175.92€ | |
| *Specialist* |  | 34 | 100 |  | 0.772 | 1.086 |  | *Outpatient clinic visit, weighted average* | 98.41€ |  | 75.96€ | 106.85€ | |
| *Nurse and other* |  | 36 | 100 |  | 0.817 | 1.086 |  | *Nursing and care, incl. Day care, per day* | 181.69€ |  | 18.56€ | 24.66€ | |
| *Total* |  | 189 | 654 |  | 4.291 | 7.101 |  |  |  |  |  |  | |
| ***Home visits*** |  |  |  |  |  |  |  |  |  |  |  |  | |
| *Family practitioner* |  | 42 | 162 |  | 0.953 | 1.759 |  | *GP visit home* | 54.07€ |  | 51.56€ | 95.11€ | |
| *Specialist* |  | 0 | 1 |  | 0.000 | 0.011 |  | *Consult doctor* | 71.38€ |  | 0€ | 0.77€ | |
| *Nurse and other* |  | 27 | 128 |  | 0.613 | 1.390 |  | *Nursing home* | 78.95€ |  | 48.39€ | 109.72€ | |
| *Total* |  | 69 | 291 |  | 1.566 | 3.159 |  |  |  |  |  |  | |
| ***All f2f patient contacts*** |  |  |  |  |  |  |  |  |  |  |  |  | |
| *Total* |  | 300 | 1115 |  | 6.811 | 12.106 |  |  |  |  |  |  | |
| ***Telephone calls*** |  |  |  |  |  |  |  |  |  |  |  |  | |
| *Total* |  | 90 | 1180 |  | 2.043 | 12.812 |  | *GP call* | 18.38€ |  | 37.56€ | 235.54€ | |
| ***All patient contacts*** |  |  |  |  |  |  |  |  |  |  |  |  | |
| *Total contacts* |  | 390 | 2295 |  | 8.854 | 24.917 |  |  |  |  |  |  | |
|  |  |  |  |  |  |  |  |  |  |  |  |  | |
| **Total** |  |  |  |  |  |  |  |  |  |  | **379.32€** | **919.09€** | |
| **Total (excluding office visits)** |  |  |  |  |  |  |  |  |  |  | **188.38€** | **623.61€** | |

Table S3 – Average hospitalisation costs for HF patients

|  | **Men** | **Women** |
| --- | --- | --- |
| % in TEN-HMS trial [17] | 78% | 22% |
| Days in hospital [20] | 8.6 | 8.4 |
| Days in hospital (average, weighted) | 8.56 | |
| Cost of a day in hospital [19] | 514.78€ | |
| **Total cost of hospitalisation (per event)** | **4,404.46€** | |

Table S4 – Average costs for HTM system and for alarm management

|  | **2015** | | | **2020 (inflation rate = 107.5%)** | | |
| --- | --- | --- | --- | --- | --- | --- |
|  | **Min** | **Mean** | **Max** | **Min** | **Mean** | **Max** |
| *Equipment and service fee (per year) [21]* | 800.00€ | 1,150.00€ | 1,500.00€ | 860.00€ | 1,236.25€ | 1,612.50€ |
| *Instalment fee (every 5 years) [21]* | 50.00€ | 100.00€ | 150.00€ | 53.75€ | 107.50€ | 161.25€ |
| ***Total HTM costs (per year)*** | **810.00€** | **1,170.00€** | **1,530.00€** | **870.75€** | **1,257.75€** | **1,644.75€** |
| **Cost of managing an alarm (per alarm) [19]** |  |  |  |  | **18.38€** |  |

Table S5 – Drug costs in the HF patient population

| **Drug** | **n [17]** | **% of total patients** | **Units** | **Formulation** | **Dosage** | **Daily dose assumption** | **HPK omschrijving [22]** | **Apotheekinkoopprijs excl. BTW [22]** | **Units [22]** | **Unit cost** | **Average monthly cost of therapy** | **Average monthly cost per patient** | **Average yearly cost per patient** | **Source [22, 26]** |
| --- | --- | --- | --- | --- | --- | --- | --- | --- | --- | --- | --- | --- | --- | --- |
| **CV drugs** | | | | | | | | | | | | | | |
| **ACE inhibitors** | | | | | | | | | | | | | | |
| Captopril | 47 | 11.0% | 3 | tabs | 25mg | 3 tabs 25mg | CAPTOPRIL MYLAN TABLET 25MG | 3.41€ | 100 | 0.0341€ | 3.28€ | 0.36€ | 4.34€ | Z index 2019 |
| Enalapril | 62 | 14.6% | 2 | tabs | 10 mg | 2 tabs 10 mg | ENALAPRIL MALEAAT MYLAN TABLET 10MG | 0.85€ | 30 | 0.0283€ | 2.16€ | 0.32€ | 3.78€ | Z index 2019 |
| Fosinopril | 44 | 10.3% | 2 | tabs | 10 mg | 2 tabs 10 mg | FOSINOPRILNATRIUM AUROBINDO TABLET 10MG | 5.75€ | 98 | 0.0587€ | 3.66€ | 0.38€ | 4.54€ | Z index 2019 |
| Lisinopril | 52 | 12.2% | 1 | tab | 10 mg | 1 tab 10 mg | LISINOPRIL AUROBINDO TABLET 10MG | 1.89€ | 100 | 0.0189€ | 0.64€ | 0.08€ | 0.93€ | Z index 2019 |
| Lisinopril | 52 | 12.2% | 1 | tab | 20 mg | 1 tab 20 mg | LISINOPRIL AUROBINDO TABLET 20MG | 2.15€ | 100 | 0.0215€ | 0.71€ | 0.09€ | 1.05€ | Z index 2019 |
| Quinapril | 32 | 7.5% | 2 | tabs | 5 mg | 2 tabs 5 mg | QUINAPRIL AUROBINDO TABLET 5MG | 1.96€ | 30 | 0.0653€ | 4.38€ | 0.33€ | 3.95€ | Z index 2019 |
| Ramipril | 107 | 25.1% | 2 | tabs | 5 mg | 2 tabs 5 mg | RAMIPRIL AUROBINDO TABLET 5MG | 4.12€ | 100 | 0.0412€ | 2.61€ | 0.66€ | 7.87€ | Z index 2019 |
| **ADP antagonists** | | | | | | | | | | | | | | |
| Clopidogrel | 25 | 5.9% | 1 | tab | 75 mg | 1 tab 75 mg | CLOPIDOGREL MYLAN TABLET FILMOMHULD 75MG | 1.13€ | 28 | 0.0404€ | 1.46€ | 0.09€ | 1.03€ | Z index 2019 |
| **Antiarrhythmics** | | | | | | | | | | | | | | |
| Amiodarone | 86 | 20.2% | 1 | tab | 200 mg | 1 tab 200 mg | AMIODARON HCL AUROBINDO TABLET 200MG | 6.88€ | 30 | 0.2293€ | 7.11€ | 1.43€ | 17.22€ | Z index 2019 |
| Digoxin | 182 | 42.7% | 1 | tab | 0.0625 mg | 1 tab 0.0625 mg | LANOXIN PG TABLET 0,0625MG | 1.20€ | 50 | 0.0240€ | 0.86€ | 0.37€ | 4.41€ | Z index 2019 |
| Digoxin | 182 | 42.7% | 1 | tab | 0.125 mg | 1 tab 0.125 mg | LANOXIN TABLET 0,125MG | 3.27€ | 60 | 0.0545€ | 1.75€ | 0.75€ | 8.97€ | Z index 2019 |
| **Anticoagulants** | | | | | | | | | | | | | | |
| Acenocoumarol | 90 | 21.1% | 4.5 | tabs | 1 mg | 4.5 tabs 1 mg | ACENOCOUMAROL CF TABLET 1MG | 1.68€ | 100 | 0.0168€ | 2.58€ | 0.55€ | 6.54€ | Z index 2019 |
| Phenprocoumon | 71 | 16.7% | 1.25 | tabs | 3 mg | 1.25 tabs 3 mg | MARCOUMAR TABLET 3MG | 7.18€ | 100 | 0.0718€ | 2.78€ | 0.46€ | 5.55€ | Z index 2019 |
| Warfarin | 44 | 10.3% |  |  | according to INR | according to INR | Average between acenocoumarol and phenprocoumon |  |  |  | 2.68€ | 0.28€ | 3.32€ | Assumption |
| **Aspirin** | | | | | | | | | | | | | | |
| Aspirin | 137 | 32.2% | 1 | tab | 100 mg | 1 tab 100 mg | ASPIRINE PROTECT EC TABLET MSR 100MG | 1.37€ | 30 | 0.0457€ | 1.60€ | 0.52€ | 6.18€ | Z index 2019 |
| **Beta-blockers** | | | | | | | | | | | | | | |
| Bisoprolol | 55 | 12.9% | 1 | tab | 10 mg | 1 tab 10 mg | BISOPROLOLFUMARAAT AUROBINDO TABLET FILMOMH 10MG | 0.70€ | 30 | 0.0233€ | 0.93€ | 0.12€ | 1.44€ | Z index 2019 |
| Carvedilol | 117 | 27.5% | 2 | tabs | 25 mg | 2 tabs 25 mg | CARVEDILOL AUROBINDO TABLET FILMOMHULD 25MG | 1.22€ | 30 | 0.0407€ | 2.90€ | 0.80€ | 9.57€ | Z index 2019 |
| Metoprolol | 58 | 13.6% | 1 | tab | 200 mg | 1 tab 200 mg | METOPROLOLTARTRAAT CF RETARD TABLET MGA 200MG | 1.78€ | 30 | 0.0593€ | 2.01€ | 0.27€ | 3.29€ | Z index 2019 |
| **Lipid lowering drugs** | | | | | | | | | | | | | | |
| Atorvastatin | 28 | 6.6% | 1 | tab | 40 mg | 1 tab 40 mg | ATORVASTATINE RANBAXY TABLET FILMOMHULD 40MG | 2.61€ | 100 | 0.0261€ | 0.85€ | 0.06€ | 0.67€ | Z index 2019 |
| Simvastatin | 48 | 11.3% | 1 | tab | 40 mg | 1 tab 40 mg | SIMVASTATINE SANDOZ TABLET FILMOMHULD 40MG | 0.69€ | 30 | 0.0230€ | 0.92€ | 0.10€ | 1.25€ | Z index 2019 |
| **Loop diuretic** | | | | | | | | | | | | | | |
| Bumetanide | 147 | 34.5% | 0.333 | tab | 1 mg | 0.333 tab 1 mg | BUMETANIDE MYLAN TABLET 1MG | 2.62€ | 90 | 0.0291€ | 0.32€ | 0.11€ | 1.31€ | Z index 2019 |
| Bumetanide | 147 | 34.5% | 0.333 | tab | 2 mg | 0.333 tab 2 mg | BUMETANIDE MYLAN TABLET 2MG | 1.26€ | 30 | 0.0420€ | 0.50€ | 0.17€ | 2.06€ | Z index 2019 |
| Bumetanide | 147 | 34.5% | 0.333 | tab | 5 mg | 0.333 tab 5 mg | BUMETANIDE SANDOZ TABLET 5MG | 3.45€ | 30 | 0.1150€ | 1.23€ | 0.42€ | 5.07€ | Z index 2019 |
| Furosemide | 271 | 63.6% | 2 | tabs | 40 mg | 2 tabs 40 mg | FUROSEMIDE MYLAN TABLET 40MG | 11.00€ | 500 | 0.0220€ | 1.35€ | 0.86€ | 10.28€ | Z index 2019 |
| **Potassium-sparing diuretics** | | | | | | | | | | | | | | |
| Spironolactone | 252 | 59.2% | 0.5 | tabs | 25 mg | 0.5 tabs 25 mg | SPIRONOLACTON ACCORD TABLET OMHULD 25MG | 0.83€ | 30 | 0.0277€ | 0.53€ | 0.31€ | 3.77€ | Z index 2019 |
| Spironolactone | 252 | 59.2% | 0.5 | tabs | 50 mg | 0.5 tabs 50 mg | SPIRONOLACTON MYLAN TABLET 50MG | 2.29€ | 30 | 0.0763€ | 1.26€ | 0.75€ | 8.95€ | Z index 2019 |
| **Other drugs** | | | | | | | | | | | | | | |
| Allopurinol | 45 | 10.6% | 1 | tab | 300 mg | 1 tab 300 mg | ALLOPURINOL MYLAN TABLET 300MG | 30.62€ | 500 | 0.0612€ | 1.85€ | 0.20€ | 2.34€ | Z index 2019 |
| Beclomethasone | 22 | 5.2% | 4 | inhalations | 50 µg | 4 inhalations 50 µg | BECLOMETASON NEVEL TEVA NEUSSPR 50MCG/DO FL 200DO | 5.96€ | 200 | 0.0298€ | 3.71€ | 0.19€ | 2.30€ | Z index 2019 |
| Insulin | 66 | 15.5% |  |  |  |  | Mean annual total costs with degludec treatment |  |  |  | 78.69€ | 12.19€ | 146.29€ | Evans 2020 |
| Ipratropium | 22 | 5.2% | 4 | inhalations | 20 µg | 4 inhalations 20 µg | ATROVENT AEROSOL 20MCG/DO SPBS 200DO + INHALATOR | 6.00€ | 200 | 0.0300€ | 3.74€ | 0.19€ | 2.32€ | Z index 2019 |
| Lactulose | 29 | 6.8% | 15 | mL | 667 mg/mL | 15 mL 667 mg/mL | DUPHALAC STROOP 667MG/ML | 3.84€ | 1000 | 0.0038€ | 1.83€ | 0.12€ | 1.50€ | Z index 2019 |
| Omeprazole | 47 | 11.0% | 1 | caps | 40 mg | 1 caps 40 mg | OMEPRAZOL FOCUS FARMA CAPSULE MSR 40MG | 2.15€ | 90 | 0.0239€ | 0.79€ | 0.09€ | 1.05€ | Z index 2019 |
| Pantoprazole | 27 | 6.3% | 1 | caps | 40 mg | 1 caps 40 mg | PANTOPRAZOL AUROBINDO TABLET MSR 40MG | 2.23€ | 100 | 0.0223€ | 0.74€ | 0.05€ | 0.56€ | Z index 2019 |
| Prednisolone | 25 | 5.9% | 2 | tabs | 5 mg | 2 tabs 5 mg | PREDNISOLON CF TABLET 5MG | 0.96€ | 30 | 0.0320€ | 2.38€ | 0.14€ | 1.68€ | Z index 2019 |
| Salbutamol | 27 | 6.3% | 4 | inhalations | 100 µg | 4 inhalations 100 µg | SALBUTAMOL MYLAN AER CFKV 100MCG/DO SPBS 200DO+INH | 2.10€ | 200 | 0.0105€ | 1.40€ | 0.09€ | 1.06€ | Z index 2019 |
| **Total** | **426** | **100.0%** |  |  |  |  |  |  |  |  |  | **23.87€** | **286.44€** |  |

Table S6 – Burden to others by NYHA class

|  | **NYHA I** | | **NYHA II** | | **NYHA III** | | **NYHA IV** | |  |
| --- | --- | --- | --- | --- | --- | --- | --- | --- | --- |
| **Burden to others [17]** | **n** | **% of known status** | **n** | **% of known status** | **n** | **% of known status** | **n** | **% of known status** | **% day informal care assumed** |
| *Blank* |  |  | 1 |  |  |  |  |  |  |
| *No* | 59 | 74.7% | 79 | 43.4% | 66 | 51.2% | 15 | 50.0% | 0% |
| *Very little* | 2 | 2.5% | 20 | 11.0% | 15 | 11.6% | 5 | 16.7% | 2% |
| *A little* | 10 | 12.7% | 41 | 22.5% | 18 | 14.0% | 3 | 10.0% | 4% |
| *Some* | 1 | 1.3% | 30 | 16.5% | 16 | 12.4% | 4 | 13.3% | 6% |
| *A lot* | 2 | 2.5% | 7 | 3.8% | 12 | 9.3% |  | 0.0% | 8% |
| *Very much* | 5 | 6.3% | 5 | 2.7% | 2 | 1.6% | 3 | 10.0% | 10% |
| *Unknown* |  |  | 2 |  | 3 |  |  |  |  |
| *Total* | 79 | 100% | 185 | 100% | 132 | 100% | 30 | 100% |  |
| ***Total % of informal care*** | **1.47%** | | **2.69%** | | **2.43%** | | **2.53%** | |  |

Table S7 – Average costs of informal care

| **Burden to others [17]** | **n** | **% of known status** | **% day informal care assumed** | **hours of informal care giving per day** | **hours of informal care giving per week** | **hours of informal care giving per year** |
| --- | --- | --- | --- | --- | --- | --- |
| *Blank* | 1 |  |  |  |  |  |
| *No* | 219 | 52.1% | 0% | 0.00 | 0 | 0 |
| *Very little* | 42 | 10.0% | 2% | 0.32 | 2.24 | 116.88 |
| *A little* | 72 | 17.1% | 4% | 0.64 | 4.48 | 233.76 |
| *Some* | 51 | 12.1% | 6% | 0.96 | 6.72 | 350.64 |
| *A lot* | 21 | 5.0% | 8% | 1.28 | 8.96 | 467.52 |
| *Very much* | 15 | 3.6% | 10% | 1.60 | 11.2 | 584.4 |
| *Unknown* | 5 |  |  |  |  |  |
| Total | 426 | 100.0% | 2.4% | 0.38 | 2.66 | 138.59 |
| Time costs of carers, replacement per hour [19] | 15.14€ | | | | | |
| Caring day (hours) | 16 | | | | | |
| **Average total costs of informal care (per patient per time)** | | | | **5.74€** | **40.21€** | **2,098.28€** |

Table S8 – Travelling expenses

|  | **Hospital** | **Outpatient** | | |
| --- | --- | --- | --- | --- |
|  | to hospital | to GP | to nursing home | to specialist |
| Average distance (km) [19] | 7 | 1.1 | 3.7 | 7 |
| %, car | 50% | 50% | 50% | 50% |
| %, public transport | 50% | 50% | 50% | 50% |
| Car, cost per kilometre [19] | 0.21€ | 0.21€ | 0.21€ | 0.21€ |
| Car, parking fee per visit [19] | 3.24€ | 3.24€ | 3.24€ | 3.24€ |
| Public transport, cost per kilometre [19] | 0.21€ | 0.21€ | 0.21€ | 0.21€ |
| Proportion in TEN-HMS trial [17] | - | 67.97% | 15.90% | 16.13% |
| Cost per item | 4.68€ | 3.47€ | 4.00€ | 4.68€ |
| ***Total cost (per visit)*** | **4.68€** | **3.75€** | | |

Table S9 – Inputs for productivity costs calculation

| **Input for productivity costs** | | |
| --- | --- | --- |
| **Probability patients that are able to work by NYHA *** | | |
| *NYHA class* | *Men* | *Women* |
| I | 88% | 88% |
| II | 61% | 61% |
| III | 17% | 17% |
| IV | 1% | 1% |
| **Net labour participation rate (general population) ** [34]** | | |
| *Age (years)* | *Men* | *Women* |
| [15 – 20[ | 57.8% | 60.2% |
| [20 – 25[ | 71.3% | 71.5% |
| [25 – 30[ | 85.8% | 84.5% |
| [30 – 35[ | 90.2% | 82.2% |
| [35 – 40[ | 92.0% | 80.6% |
| [40 – 45[ | 89.9% | 80.7% |
| [45 – 50[ | 89.2% | 80.8% |
| [50 – 55[ | 89.0% | 78.3% |
| [55 – 60[ | 84.7% | 70.1% |
| [60 – 65] | 71.2% | 51.3% |
| **Cost per hour ** [24]** | | |
| *Age (years)* | *Men* | *Women* |
| [15 – 20[ | €7.17 | € 6.75 |
| [20 – 25[ | €13.30 | € 13.14 |
| [25 – 30[ | €17.95 | € 18.08 |
| [30 – 35[ | €21.91 | € 21.61 |
| [35 – 40[ | €25.38 | € 24.09 |
| [40 – 45[ | €28.06 | € 25.01 |
| [45 – 50[ | €29.76 | € 24.74 |
| [50 – 55[ | €30.43 | € 24.19 |
| [55 – 60[ | €30.40 | € 23.95 |
| [60 – 65] | €29.51 | € 23.75 |
| **Working hours per week, excluding overtime ** [24]** | | |
| *Age (years)* | *Men* | *Women* |
| [15 – 20[ | 14.7 | 11.7 |
| [20 – 25[ | 26.3 | 22 |
| [25 – 30[ | 34.2 | 29.5 |
| [30 – 35[ | 35.9 | 28.3 |
| [35 – 40[ | 36.1 | 26.7 |
| [40 – 45[ | 36.2 | 26.2 |
| [45 – 50[ | 36.4 | 26 |
| [50 – 55[ | 36.2 | 25.5 |
| [55 – 60[ | 35.7 | 24.7 |
| [60 – 65] | 34.2 | 23.6 |
| **Working hours per day, excluding overtime †** | | |
| *Age (years)* | *Men* | *Women* |
| [15 – 20[ | 2.10 | 1.67 |
| [20 – 25[ | 3.76 | 3.14 |
| [25 – 30[ | 4.89 | 4.21 |
| [30 – 35[ | 5.13 | 4.04 |
| [35 – 40[ | 5.16 | 3.81 |
| [40 – 45[ | 5.17 | 3.74 |
| [45 – 50[ | 5.20 | 3.71 |
| [50 – 55[ | 5.17 | 3.64 |
| [55 – 60[ | 5.10 | 3.53 |
| [60 – 65] | 4.89 | 3.37 |
| **Cost per day** | | |
| *Age (years)* | *Men* | *Women* |
| [15 – 20[ | €15.06 | € 11.28 |
| [20 – 25[ | €49.97 | € 41.29 |
| [25 – 30[ | €87.70 | € 76.20 |
| [30 – 35[ | €112.37 | € 87.36 |
| [35 – 40[ | €130.87 | € 91.88 |
| [40 – 45[ | €145.11 | € 93.61 |
| [45 – 50[ | €154.76 | € 91.88 |
| [50 – 55[ | €157.37 | € 88.12 |
| [55 – 60[ | €155.04 | € 84.50 |
| [60 – 65] | €144.17 | € 80.09 |
| * Expert opinion ** CBS (2019) † It is unknown on which day of the week patients are admitted to the hospital a correction for unproductive hours, such as holidays or sick leave, should not be applied. In this case, the number of calendar days, and not number of workdays, was used. Therefore, seven days per week (instead of 5) were assumed. | | |
|  |  |  |
|  |  |  |
|  |  |  |

Table S10 – Example of productivity costs calculation for a hypothetical patient

| **Example productivity costs calculation** | |
| --- | --- |
| Age | 50 |
| Gender | Male |
| Productivity costs per hour (age and gender dependent) [23] | € 30.43 |
| Productive hours per working day (age and gender dependent) [23] | 36.2/7= 5.17 |
| Costs per patient per day | €30.43 x 5.17 = €157.32 |
| Probability paid job (age) | 89.0% |
| Expected costs per patient per day | €155.31 x 0.89 = €140.01 |
| NYHA class | II |
| Percentage working NYHA class II | 61% |
| Expected costs per working NYHA class II patient per day | €140.01 x 0.61 = €85.41 |
| **Productivity costs per hospitalisation for this patient (one month absence)** | **€85.41 x (365.25/12) = €2599.67** |

Table S11 – Age-gender-specific per capita future unrelated medical costs for HF [27]

| **Age** | **Last year of life (men)** | **Last year of life (women)** | **Other years of life (men)** | **Other years of life (women)** |
| --- | --- | --- | --- | --- |
| 18 | 19,207€ | 19,439€ | 2,512€ | 2,517€ |
| 19 | 18,498€ | 19,691€ | 2,587€ | 2,633€ |
| 20 | 17,856€ | 20,099€ | 2,658€ | 2,755€ |
| 21 | 17,327€ | 20,711€ | 2,711€ | 2,878€ |
| 22 | 16,941€ | 21,577€ | 2,736€ | 2,993€ |
| 23 | 16,714€ | 22,716€ | 2,730€ | 3,100€ |
| 24 | 16,645€ | 24,102€ | 2,703€ | 3,205€ |
| 25 | 16,723€ | 25,690€ | 2,666€ | 3,310€ |
| 26 | 16,948€ | 27,432€ | 2,628€ | 3,417€ |
| 27 | 17,326€ | 29,239€ | 2,602€ | 3,526€ |
| 28 | 17,806€ | 31,011€ | 2,591€ | 3,632€ |
| 29 | 18,333€ | 32,626€ | 2,592€ | 3,723€ |
| 30 | 18,878€ | 33,994€ | 2,602€ | 3,790€ |
| 31 | 19,451€ | 35,055€ | 2,619€ | 3,829€ |
| 32 | 20,076€ | 35,773€ | 2,641€ | 3,836€ |
| 33 | 20,775€ | 36,177€ | 2,667€ | 3,813€ |
| 34 | 21,551€ | 36,368€ | 2,695€ | 3,764€ |
| 35 | 22,388€ | 36,453€ | 2,724€ | 3,696€ |
| 36 | 23,267€ | 36,512€ | 2,751€ | 3,614€ |
| 37 | 24,169€ | 36,628€ | 2,776€ | 3,525€ |
| 38 | 25,086€ | 36,861€ | 2,799€ | 3,437€ |
| 39 | 26,015€ | 37,235€ | 2,823€ | 3,358€ |
| 40 | 26,963€ | 37,767€ | 2,850€ | 3,295€ |
| 41 | 27,944€ | 38,509€ | 2,883€ | 3,258€ |
| 42 | 28,988€ | 39,532€ | 2,925€ | 3,253€ |
| 43 | 30,121€ | 40,843€ | 2,978€ | 3,280€ |
| 44 | 31,361€ | 42,376€ | 3,039€ | 3,330€ |
| 45 | 32,727€ | 44,037€ | 3,107€ | 3,398€ |
| 46 | 34,237€ | 45,809€ | 3,182€ | 3,476€ |
| 47 | 35,907€ | 47,724€ | 3,264€ | 3,562€ |
| 48 | 37,742€ | 49,874€ | 3,352€ | 3,650€ |
| 49 | 39,707€ | 52,282€ | 3,444€ | 3,740€ |
| 50 | 41,649€ | 54,675€ | 3,538€ | 3,829€ |
| 51 | 43,472€ | 56,809€ | 3,634€ | 3,915€ |
| 52 | 45,108€ | 58,432€ | 3,730€ | 3,994€ |
| 53 | 46,558€ | 59,503€ | 3,828€ | 4,067€ |
| 54 | 47,844€ | 60,146€ | 3,930€ | 4,137€ |
| 55 | 49,018€ | 60,547€ | 4,038€ | 4,206€ |
| 56 | 50,128€ | 60,874€ | 4,154€ | 4,278€ |
| 57 | 51,142€ | 61,079€ | 4,281€ | 4,356€ |
| 58 | 51,980€ | 61,044€ | 4,419€ | 4,441€ |
| 59 | 52,533€ | 60,643€ | 4,565€ | 4,532€ |
| 60 | 52,766€ | 59,876€ | 4,716€ | 4,630€ |
| 61 | 52,808€ | 58,985€ | 4,871€ | 4,739€ |
| 62 | 52,808€ | 58,220€ | 5,028€ | 4,859€ |
| 63 | 52,823€ | 57,675€ | 5,189€ | 4,996€ |
| 64 | 52,852€ | 57,307€ | 5,356€ | 5,149€ |
| 65 | 52,841€ | 56,979€ | 5,530€ | 5,318€ |
| 66 | 52,745€ | 56,568€ | 5,713€ | 5,502€ |
| 67 | 52,540€ | 55,989€ | 5,908€ | 5,699€ |
| 68 | 52,245€ | 55,272€ | 6,120€ | 5,916€ |
| 69 | 51,897€ | 54,502€ | 6,355€ | 6,162€ |
| 70 | 51,504€ | 53,764€ | 6,617€ | 6,446€ |
| 71 | 51,079€ | 53,128€ | 6,913€ | 6,780€ |
| 72 | 50,670€ | 52,637€ | 7,249€ | 7,173€ |
| 73 | 50,325€ | 52,329€ | 7,630€ | 7,632€ |
| 74 | 50,075€ | 52,219€ | 8,053€ | 8,160€ |
| 75 | 49,866€ | 52,288€ | 8,516€ | 8,761€ |
| 76 | 49,593€ | 52,487€ | 9,010€ | 9,435€ |
| 77 | 49,159€ | 52,767€ | 9,528€ | 10,184€ |
| 78 | 48,549€ | 53,154€ | 10,071€ | 11,025€ |
| 79 | 47,916€ | 53,723€ | 10,663€ | 11,985€ |
| 80 | 47,391€ | 54,519€ | 11,337€ | 13,095€ |
| 81 | 47,138€ | 55,608€ | 12,123€ | 14,383€ |
| 82 | 47,191€ | 57,025€ | 13,054€ | 15,878€ |
| 83 | 47,439€ | 58,657€ | 14,127€ | 17,573€ |
| 84 | 47,807€ | 60,399€ | 15,306€ | 19,419€ |
| 85 | 48,348€ | 62,296€ | 16,538€ | 21,353€ |
| 86 | 48,966€ | 64,231€ | 17,795€ | 23,329€ |
| 87 | 49,665€ | 66,129€ | 19,085€ | 25,319€ |
| 88 | 50,590€ | 68,086€ | 20,465€ | 27,352€ |
| 89 | 51,867€ | 70,189€ | 21,999€ | 29,465€ |
| 90 | 53,530€ | 72,457€ | 23,729€ | 31,685€ |
| 91 | 55,554€ | 74,860€ | 25,683€ | 34,035€ |
| 92 | 57,837€ | 77,295€ | 27,871€ | 36,526€ |
| 93 | 60,133€ | 79,575€ | 30,251€ | 39,147€ |
| 94 | 62,256€ | 81,618€ | 32,830€ | 41,965€ |
| 95 | 64,107€ | 83,482€ | 35,649€ | 45,088€ |
| 96 | 65,395€ | 84,859€ | 38,938€ | 48,813€ |
| 97 | 61,376€ | 79,794€ | 39,895€ | 49,948€ |
| 98 | 57,096€ | 73,885€ | 41,508€ | 51,534€ |
| 99 | 52,664€ | 67,295€ | 44,106€ | 53,738€ |

Table S12 – Utility values per NYHA class [21]

| **NYHA class** | **Mean** | **SE** |
| --- | --- | --- |
| I | 0.87976 | 0.00827 |
| II | 0.71178 | 0.00944 |
| III | 0.61405 | 0.01349 |
| IV | 0.49228 | 0.03032 |
| NYHA, New York Heart Association; SE, standard error. | | |

Equation S1 – Utility for the general population [28]

$$Utility=0.9508566+0.0212126\times male-0.0002587\times age-0.0000332\times{age}^{2}$$

Table S13 – Scenario analyses: summary of cost-effectiveness results

|  | **Area of uncertainty** | **Scenario** | **Costs (€)** | | | **QALYs** | | | **ICER (€/QALY)** | |
| --- | --- | --- | --- | --- | --- | --- | --- | --- | --- | --- |
|  |  |  | **UC** | **HTM*** | **HTM+DA** | **UC** | **HTM*** | **HTM+DA** | **HTM+DA vs. UC** | **% vs. base-case** |
| - | N/A | Base-case | €46,879 | €60,343 | €65,008 | 1.12 | 1.51 | 1.78 | €27,712 | 0% |
| 1 | Time-to-death | Parametric model: exponential | €43,562 | €56,841 | €61,171 | 1.01 | 1.40 | 1.66 | €26,841 | -3.1% |
| 2 |  | Parametric model: log-normal | €47,273 | €67,213 | €74,934 | 1.17 | 1.75 | 2.13 | €28,899 | +4.3% |
| 3 |  | Parametric model: log-logistic | €47,730 | €63,808 | €69,023 | 1.18 | 1.62 | 1.93 | €28,370 | +2.4% |
| 4 |  | Parametric model: Gompertz | €44,584 | €57,938 | €61,947 | 1.04 | 1.44 | 1.69 | €27,060 | -2.4% |
| 5 |  | Parametric model: generalised gamma | €47,250 | €63,982 | €70,247 | 1.16 | 1.63 | 1.96 | €28,651 | +3.4% |
| 6 | Time-to-hospitalisation | Parametric model: exponential | €47,287 | €63,040 | €66,624 | 1.26 | 1.70 | 1.94 | €28,440 | +2.6% |
| 7 |  | Parametric model: Weibull | €47,360 | €61,139 | €65,248 | 1.17 | 1.49 | 1.75 | €31,062 | +12.1% |
| 8 |  | Parametric model: log-logistic | €46,907 | €61,063 | €65,803 | 1.17 | 1.56 | 1.82 | €28,951 | +4.5% |
| 9 |  | Parametric model: Gompertz | €47,268 | €60,976 | €65,174 | 1.16 | 1.50 | 1.78 | €28,950 | +4.5% |
| 10 |  | Parametric model: generalised gamma | €46,717 | €60,778 | €64,945 | 1.14 | 1.53 | 1.79 | €28,084 | +1.3% |
| 11 | Time-to-outpatient visit | Set to every 6 months for all interventions | €44,746 | €58,038 | €61,784 | 1.13 | 1.48 | 1.74 | €27,621 | -0.3% |
| 12 |  | Set to every 1 year for all interventions | €43,864 | €58,916 | €63,191 | 1.21 | 1.62 | 1.90 | €28,104 | +1.4% |
| 13 | Utility | No utility decrement from hospitalisation | €46,879 | €60,343 | €64,521 | 1.52 | 2.06 | 2.37 | €20,687 | -25.3% |
| 14 |  | All utilities from lower bound of 95% confidence intervals | €46,879 | €60,343 | €64,521 | 1.10 | 1.48 | 1.74 | €27,463 | -0.9% |
| 15 |  | All utilities from upper bound of 95% confidence intervals | €46,879 | €60,343 | €64,521 | 1.15 | 1.54 | 1.81 | €26,442 | -4.6% |
| 16 | Costs | [Drug costs from expert opinion from literature](file:///C:\Users\Fernando\Desktop\Scenario_subgroup_analyses.xlsx#RANGE!_ENREF_11) [25] | €49,592 | €63,951 | €68,647 | 1.12 | 1.51 | 1.78 | €29,093 | +5.0% |
| 17 |  | No costs for informal care | €42,588 | €54,640 | €57,997 | 1.12 | 1.51 | 1.78 | €23,528 | -15.1% |
| 18 |  | No productivity costs | €44,698 | €57,859 | €62,284 | 1.12 | 1.51 | 1.78 | €26,852 | -3.1% |
| 19 |  | No costs for device | €46,879 | €56,924 | €60,610 | 1.12 | 1.51 | 1.78 | €20,966 | -24.3% |
| 20 |  | No future unrelated medical costs | €22,548 | €33,705 | €33,784 | 1.12 | 1.51 | 1.78 | €17,155 | -38.1% |
| 21 |  | All costs from lower bound of 95% confidence intervals | €40,673 | €51,334 | €55,485 | 1.12 | 1.51 | 1.78 | €22,616 | -18.4% |
| 22 |  | All costs from upper bound of 95% confidence intervals | €54,009 | €70,653 | €74,855 | 1.12 | 1.51 | 1.78 | €31,829 | +14.9% |
| 23 |  | Healthcare perspective (no productivity, informal care, travel, and future unrelated medical costs) | €16,034 | €25,433 | €25,460 | 1.12 | 1.51 | 1.78 | €14,408 | -48.0% |
| * HTM is extendedly dominated by HTM+DA in all scenarios. The ICER comparison against the base-case is only shown for HTM+DA vs. UC. | | | | | | | | | | |
