# Supplementary material for: Home Telemonitoring and a Diagnostic Algorithm in the Management of Heart Failure in the Netherlands: Cost-effectiveness Analysis
Source: JMIR Cardio. 2022 Aug 4;6(2):e31302. doi: 10.2196/31302 (PMC9389378; doi:10.2196/31302)
Supplement: Multimedia Appendix 2 [file cardio_v6i2e31302_app2.docx]

**Supplementary material II**

(For the manuscript: Cost-effectiveness of a home telemonitoring system and a diagnostic algorithm in the management of heart failure in the Netherlands)

Table S1 – Baseline patient and disease characteristics of the model population for subgroup 1 (age < 65 years old)

|  | Baseline characteristics of the starting population |
| --- | --- |
| Sample size | 136 |
| Ejection fraction (EF), % (mean) | 24.17 |
| Age, years (mean) | 53.85 |
| Systolic blood pressure (SBP), mm Hg (mean) | 108.16 |
| Body mass index (BMI), kg/m2 (mean) | 26.97 |
| Creatinine, µmol/l (mean) | 119.57 |
| NYHA class 1, % | 24.1 |
| NYHA class 2, % | 38.8 |
| NYHA class 3, % | 32.7 |
| NYHA class 4, % | 4.4 |
| Gender (male), % | 79.3 |
| Smoker, % | 17.8 |
| Diabetes, % | 32.6 |
| Chronic obstructive pulmonary disease (COPD), % | 21.2 |
| Recent diagnosis, % | 46.4 |
| No beta-blocker medication, % | 36.5 |
| No ACE inhibitor medication, % | 14.0 |
| Myocardial infarction, % | 46.0 |
| Chronic atrial fibrillation, % | 10.0 |
| ACE, angiotensin-converting enzyme; BMI, body mass index; COPD, chronic obstructive pulmonary disease; EF, ejection fraction; NYHA, New York Heart Association; SBP, systolic blood pressure. | |

Table S2 – Baseline patient and disease characteristics of the model population for subgroup 2 (age ≥ 65 years old)

|  | Baseline characteristics of the starting population |
| --- | --- |
| Sample size | 290 |
| Ejection fraction (EF), % (mean) | 25.23 |
| Age, years (mean) | 73.74 |
| Systolic blood pressure (SBP), mm Hg (mean) | 116.58 |
| Body mass index (BMI), kg/m2 (mean) | 25.58 |
| Creatinine, µmol/l (mean) | 143.65 |
| NYHA class 1, % | 15.8 |
| NYHA class 2, % | 45.3 |
| NYHA class 3, % | 30.2 |
| NYHA class 4, % | 8.7 |
| Gender (male), % | 75.1 |
| Smoker, % | 8.6 |
| Diabetes, % | 37.4 |
| Chronic obstructive pulmonary disease (COPD), % | 25.9 |
| Recent diagnosis, % | 43.4 |
| No beta-blocker medication, % | 35.8 |
| No ACE inhibitor medication, % | 20.5 |
| Myocardial infarction, % | 62.4 |
| Chronic atrial fibrillation, % | 35.2 |
| ACE, angiotensin-converting enzyme; BMI, body mass index; COPD, chronic obstructive pulmonary disease; EF, ejection fraction; NYHA, New York Heart Association; SBP, systolic blood pressure. | |

Table S3 – Baseline patient and disease characteristics of the model population for subgroup 3 (ejection fraction < 25%)

|  | Baseline characteristics of the starting population |
| --- | --- |
| Sample size | 214 |
| Ejection fraction (EF), % (mean) | 18.89 |
| Age, years (mean) | 67.19 |
| Systolic blood pressure (SBP), mm Hg (mean) | 111.59 |
| Body mass index (BMI), kg/m2 (mean) | 25.05 |
| Creatinine, µmol/l (mean) | 132.21 |
| NYHA class 1, % | 20.9 |
| NYHA class 2, % | 44.6 |
| NYHA class 3, % | 28.9 |
| NYHA class 4, % | 5.6 |
| Gender (male), % | 77.0 |
| Smoker, % | 14.1 |
| Diabetes, % | 25.3 |
| Chronic obstructive pulmonary disease (COPD), % | 21.8 |
| Recent diagnosis, % | 48.8 |
| No beta-blocker medication, % | 31.2 |
| No ACE inhibitor medication, % | 14.7 |
| Myocardial infarction, % | 58.1 |
| Chronic atrial fibrillation, % | 23.7 |
| ACE, angiotensin-converting enzyme; BMI, body mass index; COPD, chronic obstructive pulmonary disease; EF, ejection fraction; NYHA, New York Heart Association; SBP, systolic blood pressure. | |

Table S4 – Baseline patient and disease characteristics of the model population for subgroup 4 (ejection fraction ≥ 25%)

|  | Baseline characteristics of the starting population |
| --- | --- |
| Sample size | 212 |
| Ejection fraction (EF), % (mean) | 31.44 |
| Age, years (mean) | 68.19 |
| Systolic blood pressure (SBP), mm Hg (mean) | 116.72 |
| Body mass index (BMI), kg/m2 (mean) | 26.89 |
| Creatinine, µmol/l (mean) | 139.57 |
| NYHA class 1, % | 16.2 |
| NYHA class 2, % | 45.2 |
| NYHA class 3, % | 29.8 |
| NYHA class 4, % | 8.8 |
| Gender (male), % | 76.4 |
| Smoker, % | 9.1 |
| Diabetes, % | 38.8 |
| Chronic obstructive pulmonary disease (COPD), % | 24.9 |
| Recent diagnosis, % | 41.5 |
| No beta-blocker medication, % | 42.2 |
| No ACE inhibitor medication, % | 23.2 |
| Myocardial infarction, % | 58.1 |
| Chronic atrial fibrillation, % | 27.7 |
| ACE, angiotensin-converting enzyme; BMI, body mass index; COPD, chronic obstructive pulmonary disease; EF, ejection fraction; NYHA, New York Heart Association; SBP, systolic blood pressure. | |

Table S5 – Baseline patient and disease characteristics of the model population for subgroup 5 (NYHA class I)

|  | Baseline characteristics of the starting population |
| --- | --- |
| Sample size | 79 |
| Ejection fraction (EF), % (mean) | 24.00 |
| Age, years (mean) | 66.32 |
| Systolic blood pressure (SBP), mm Hg (mean) | 111.35 |
| Body mass index (BMI), kg/m2 (mean) | 25.21 |
| Creatinine, µmol/l (mean) | 130.03 |
| NYHA class 1, % | 100.0 |
| NYHA class 2, % | 0.0 |
| NYHA class 3, % | 0.0 |
| NYHA class 4, % | 0.0 |
| Gender (male), % | 84.9 |
| Smoker, % | 17.6 |
| Diabetes, % | 28.0 |
| Chronic obstructive pulmonary disease (COPD), % | 23.8 |
| Recent diagnosis, % | 52.8 |
| No beta-blocker medication, % | 28.0 |
| No ACE inhibitor medication, % | 4.1 |
| Myocardial infarction, % | 59.4 |
| Chronic atrial fibrillation, % | 14.3 |
| ACE, angiotensin-converting enzyme; BMI, body mass index; COPD, chronic obstructive pulmonary disease; EF, ejection fraction; NYHA, New York Heart Association; SBP, systolic blood pressure. | |

Table S6 – Baseline patient and disease characteristics of the model population for subgroup 6 (NYHA class II)

|  | Baseline characteristics of the starting population |
| --- | --- |
| Sample size | 185 |
| Ejection fraction (EF), % (mean) | 24.56 |
| Age, years (mean) | 67.46 |
| Systolic blood pressure (SBP), mm Hg (mean) | 117.41 |
| Body mass index (BMI), kg/m2 (mean) | 26.24 |
| Creatinine, µmol/l (mean) | 133.88 |
| NYHA class 1, % | 0.0 |
| NYHA class 2, % | 100.0 |
| NYHA class 3, % | 0.0 |
| NYHA class 4, % | 0.0 |
| Gender (male), % | 75.1 |
| Smoker, % | 13.6 |
| Diabetes, % | 33.9 |
| Chronic obstructive pulmonary disease (COPD), % | 27.9 |
| Recent diagnosis, % | 46.7 |
| No beta-blocker medication, % | 31.4 |
| No ACE inhibitor medication, % | 18.6 |
| Myocardial infarction, % | 56.9 |
| Chronic atrial fibrillation, % | 28.0 |
| ACE, angiotensin-converting enzyme; BMI, body mass index; COPD, chronic obstructive pulmonary disease; EF, ejection fraction; NYHA, New York Heart Association; SBP, systolic blood pressure. | |

Table S7 – Baseline patient and disease characteristics of the model population for subgroup 7 (NYHA class III)

|  | Baseline characteristics of the starting population |
| --- | --- |
| Sample size | 132 |
| Ejection fraction (EF), % (mean) | 25.62 |
| Age, years (mean) | 67.92 |
| Systolic blood pressure (SBP), mm Hg (mean) | 114.62 |
| Body mass index (BMI), kg/m2 (mean) | 26.39 |
| Creatinine, µmol/l (mean) | 141.95 |
| NYHA class 1, % | 0.0 |
| NYHA class 2, % | 0.0 |
| NYHA class 3, % | 100.0 |
| NYHA class 4, % | 0.0 |
| Gender (male), % | 74.7 |
| Smoker, % | 6.4 |
| Diabetes, % | 36.3 |
| Chronic obstructive pulmonary disease (COPD), % | 18.7 |
| Recent diagnosis, % | 37.9 |
| No beta-blocker medication, % | 41.0 |
| No ACE inhibitor medication, % | 24.1 |
| Myocardial infarction, % | 54.1 |
| Chronic atrial fibrillation, % | 29.5 |
| ACE, angiotensin-converting enzyme; BMI, body mass index; COPD, chronic obstructive pulmonary disease; EF, ejection fraction; NYHA, New York Heart Association; SBP, systolic blood pressure. | |

Table S8 – Baseline patient and disease characteristics of the model population for subgroup 8 (NYHA class IV)

|  | Baseline characteristics of the starting population |
| --- | --- |
| Sample size | 30 |
| Ejection fraction (EF), % (mean) | 27.13 |
| Age, years (mean) | 70.58 |
| Systolic blood pressure (SBP), mm Hg (mean) | 112.16 |
| Body mass index (BMI), kg/m2 (mean) | 26.12 |
| Creatinine, µmol/l (mean) | 137.50 |
| NYHA class 1, % | 0.0 |
| NYHA class 2, % | 0.0 |
| NYHA class 3, % | 0.0 |
| NYHA class 4, % | 100.0 |
| Gender (male), % | 88.9 |
| Smoker, % | 2.6 |
| Diabetes, % | 35.5 |
| Chronic obstructive pulmonary disease (COPD), % | 22.8 |
| Recent diagnosis, % | 34.1 |
| No beta-blocker medication, % | 60.1 |
| No ACE inhibitor medication, % | 30.9 |
| Myocardial infarction, % | 73.7 |
| Chronic atrial fibrillation, % | 33.3 |
| ACE, angiotensin-converting enzyme; BMI, body mass index; COPD, chronic obstructive pulmonary disease; EF, ejection fraction; NYHA, New York Heart Association; SBP, systolic blood pressure. | |

Table S9 – Baseline patient and disease characteristics of the model population for subgroup 9 (gender: male)

|  | Baseline characteristics of the starting population |
| --- | --- |
| Sample size | 330 |
| Ejection fraction (EF), % (mean) | 25.03 |
| Age, years (mean) | 67.07 |
| Systolic blood pressure (SBP), mm Hg (mean) | 113.25 |
| Body mass index (BMI), kg/m2 (mean) | 26.29 |
| Creatinine, µmol/l (mean) | 135.82 |
| NYHA class 1, % | 18.4 |
| NYHA class 2, % | 40.1 |
| NYHA class 3, % | 32.2 |
| NYHA class 4, % | 9.3 |
| Gender (male), % | 100.0 |
| Smoker, % | 11.3 |
| Diabetes, % | 32.8 |
| Chronic obstructive pulmonary disease (COPD), % | 23.9 |
| Recent diagnosis, % | 42.6 |
| No beta-blocker medication, % | 39.8 |
| No ACE inhibitor medication, % | 18.9 |
| Myocardial infarction, % | 60.9 |
| Chronic atrial fibrillation, % | 28.4 |
| ACE, angiotensin-converting enzyme; BMI, body mass index; COPD, chronic obstructive pulmonary disease; EF, ejection fraction; NYHA, New York Heart Association; SBP, systolic blood pressure. | |

Table S10 – Baseline patient and disease characteristics of the model population for subgroup 10 (gender: female)

|  | Baseline characteristics of the starting population |
| --- | --- |
| Sample size | 96 |
| Ejection fraction (EF), % (mean) | 24.78 |
| Age, years (mean) | 68.78 |
| Systolic blood pressure (SBP), mm Hg (mean) | 117.50 |
| Body mass index (BMI), kg/m2 (mean) | 26.23 |
| Creatinine, µmol/l (mean) | 126.16 |
| NYHA class 1, % | 16.0 |
| NYHA class 2, % | 46.9 |
| NYHA class 3, % | 34.6 |
| NYHA class 4, % | 2.5 |
| Gender (male), % | 0.0 |
| Smoker, % | 10.9 |
| Diabetes, % | 36.5 |
| Chronic obstructive pulmonary disease (COPD), % | 24.9 |
| Recent diagnosis, % | 44.9 |
| No beta-blocker medication, % | 31.9 |
| No ACE inhibitor medication, % | 16.6 |
| Myocardial infarction, % | 47.9 |
| Chronic atrial fibrillation, % | 18.1 |
| ACE, angiotensin-converting enzyme; BMI, body mass index; COPD, chronic obstructive pulmonary disease; EF, ejection fraction; NYHA, New York Heart Association; SBP, systolic blood pressure. | |

Table S11 – Baseline patient and disease characteristics of the model population for subgroup 11 (smoker: yes)

|  | Baseline characteristics of the starting population |
| --- | --- |
| Sample size | 52 |
| Ejection fraction (EF), % (mean) | 24.26 |
| Age, years (mean) | 65.54 |
| Systolic blood pressure (SBP), mm Hg (mean) | 118.47 |
| Body mass index (BMI), kg/m2 (mean) | 26.38 |
| Creatinine, µmol/l (mean) | 126.19 |
| NYHA class 1, % | 22.6 |
| NYHA class 2, % | 54.7 |
| NYHA class 3, % | 21.0 |
| NYHA class 4, % | 1.7 |
| Gender (male), % | 82.5 |
| Smoker, % | 100.0 |
| Diabetes, % | 27.6 |
| Chronic obstructive pulmonary disease (COPD), % | 42.4 |
| Recent diagnosis, % | 49.8 |
| No beta-blocker medication, % | 31.0 |
| No ACE inhibitor medication, % | 12.2 |
| Myocardial infarction, % | 44.2 |
| Chronic atrial fibrillation, % | 14.8 |
| ACE, angiotensin-converting enzyme; BMI, body mass index; COPD, chronic obstructive pulmonary disease; EF, ejection fraction; NYHA, New York Heart Association; SBP, systolic blood pressure. | |

Table S12 – Baseline patient and disease characteristics of the model population for subgroup 12 (smoker: no)

|  | Baseline characteristics of the starting population |
| --- | --- |
| Sample size | 374 |
| Ejection fraction (EF), % (mean) | 25.35 |
| Age, years (mean) | 67.52 |
| Systolic blood pressure (SBP), mm Hg (mean) | 114.05 |
| Body mass index (BMI), kg/m2 (mean) | 26.27 |
| Creatinine, µmol/l (mean) | 136.89 |
| NYHA class 1, % | 16.3 |
| NYHA class 2, % | 45.6 |
| NYHA class 3, % | 31.4 |
| NYHA class 4, % | 6.7 |
| Gender (male), % | 78.3 |
| Smoker, % | 0.0 |
| Diabetes, % | 37.3 |
| Chronic obstructive pulmonary disease (COPD), % | 21.0 |
| Recent diagnosis, % | 42.2 |
| No beta-blocker medication, % | 38.1 |
| No ACE inhibitor medication, % | 19.2 |
| Myocardial infarction, % | 60.9 |
| Chronic atrial fibrillation, % | 27.9 |
| ACE, angiotensin-converting enzyme; BMI, body mass index; COPD, chronic obstructive pulmonary disease; EF, ejection fraction; NYHA, New York Heart Association; SBP, systolic blood pressure. | |

Table S13 – Baseline patient and disease characteristics of the model population for subgroup 13 (diabetes: yes)

|  | Baseline characteristics of the starting population |
| --- | --- |
| Sample size | 149 |
| Ejection fraction (EF), % (mean) | 26.31 |
| Age, years (mean) | 68.65 |
| Systolic blood pressure (SBP), mm Hg (mean) | 117.83 |
| Body mass index (BMI), kg/m2 (mean) | 27.23 |
| Creatinine, µmol/l (mean) | 133.44 |
| NYHA class 1, % | 15.6 |
| NYHA class 2, % | 42.0 |
| NYHA class 3, % | 34.6 |
| NYHA class 4, % | 7.8 |
| Gender (male), % | 77.0 |
| Smoker, % | 9.6 |
| Diabetes, % | 100.0 |
| Chronic obstructive pulmonary disease (COPD), % | 21.7 |
| Recent diagnosis, % | 42.8 |
| No beta-blocker medication, % | 33.0 |
| No ACE inhibitor medication, % | 15.7 |
| Myocardial infarction, % | 67.2 |
| Chronic atrial fibrillation, % | 29.1 |
| ACE, angiotensin-converting enzyme; BMI, body mass index; COPD, chronic obstructive pulmonary disease; EF, ejection fraction; NYHA, New York Heart Association; SBP, systolic blood pressure. | |

Table S14 – Baseline patient and disease characteristics of the model population for subgroup 14 (diabetes: no)

|  | Baseline characteristics of the starting population |
| --- | --- |
| Sample size | 277 |
| Ejection fraction (EF), % (mean) | 24.50 |
| Age, years (mean) | 67.19 |
| Systolic blood pressure (SBP), mm Hg (mean) | 112.49 |
| Body mass index (BMI), kg/m2 (mean) | 25.51 |
| Creatinine, µmol/l (mean) | 139.72 |
| NYHA class 1, % | 19.0 |
| NYHA class 2, % | 43.8 |
| NYHA class 3, % | 30.3 |
| NYHA class 4, % | 6.9 |
| Gender (male), % | 75.2 |
| Smoker, % | 12.4 |
| Diabetes, % | 0.0 |
| Chronic obstructive pulmonary disease (COPD), % | 25.3 |
| Recent diagnosis, % | 41.7 |
| No beta-blocker medication, % | 39.9 |
| No ACE inhibitor medication, % | 19.8 |
| Myocardial infarction, % | 49.1 |
| Chronic atrial fibrillation, % | 25.5 |
| ACE, angiotensin-converting enzyme; BMI, body mass index; COPD, chronic obstructive pulmonary disease; EF, ejection fraction; NYHA, New York Heart Association; SBP, systolic blood pressure. | |

Table S15 – Baseline patient and disease characteristics of the model population for subgroup 15 (COPD: yes)

|  | Baseline characteristics of the starting population |
| --- | --- |
| Sample size | 104 |
| Ejection fraction (EF), % (mean) | 25.95 |
| Age, years (mean) | 68.22 |
| Systolic blood pressure (SBP), mm Hg (mean) | 117.20 |
| Body mass index (BMI), kg/m2 (mean) | 26.35 |
| Creatinine, µmol/l (mean) | 140.33 |
| NYHA class 1, % | 19.0 |
| NYHA class 2, % | 48.5 |
| NYHA class 3, % | 25.5 |
| NYHA class 4, % | 7.0 |
| Gender (male), % | 78.5 |
| Smoker, % | 21.2 |
| Diabetes, % | 34.0 |
| Chronic obstructive pulmonary disease (COPD), % | 100.0 |
| Recent diagnosis, % | 37.4 |
| No beta-blocker medication, % | 57.2 |
| No ACE inhibitor medication, % | 20.1 |
| Myocardial infarction, % | 48.9 |
| Chronic atrial fibrillation, % | 27.3 |
| ACE, angiotensin-converting enzyme; BMI, body mass index; COPD, chronic obstructive pulmonary disease; EF, ejection fraction; NYHA, New York Heart Association; SBP, systolic blood pressure. | |

Table S16 – Baseline patient and disease characteristics of the model population for subgroup 16 (COPD: no)

|  | Baseline characteristics of the starting population |
| --- | --- |
| Sample size | 322 |
| Ejection fraction (EF), % (mean) | 25.09 |
| Age, years (mean) | 67.38 |
| Systolic blood pressure (SBP), mm Hg (mean) | 113.55 |
| Body mass index (BMI), kg/m2 (mean) | 26.05 |
| Creatinine, µmol/l (mean) | 134.34 |
| NYHA class 1, % | 17.5 |
| NYHA class 2, % | 40.4 |
| NYHA class 3, % | 34.6 |
| NYHA class 4, % | 7.5 |
| Gender (male), % | 77.9 |
| Smoker, % | 7.6 |
| Diabetes, % | 35.6 |
| Chronic obstructive pulmonary disease (COPD), % | 0.0 |
| Recent diagnosis, % | 44.8 |
| No beta-blocker medication, % | 31.4 |
| No ACE inhibitor medication, % | 20.0 |
| Myocardial infarction, % | 58.2 |
| Chronic atrial fibrillation, % | 27.0 |
| ACE, angiotensin-converting enzyme; BMI, body mass index; COPD, chronic obstructive pulmonary disease; EF, ejection fraction; NYHA, New York Heart Association; SBP, systolic blood pressure. | |

Table S17 – Baseline patient and disease characteristics of the model population for subgroup 17 (recent diagnosis: yes)

|  | Baseline characteristics of the starting population |
| --- | --- |
| Sample size | 187 |
| Ejection fraction (EF), % (mean) | 24.84 |
| Age, years (mean) | 66.56 |
| Systolic blood pressure (SBP), mm Hg (mean) | 115.36 |
| Body mass index (BMI), kg/m2 (mean) | 25.74 |
| Creatinine, µmol/l (mean) | 123.24 |
| NYHA class 1, % | 21.0 |
| NYHA class 2, % | 48.6 |
| NYHA class 3, % | 24.7 |
| NYHA class 4, % | 5.7 |
| Gender (male), % | 75.4 |
| Smoker, % | 14.8 |
| Diabetes, % | 33.9 |
| Chronic obstructive pulmonary disease (COPD), % | 22.1 |
| Recent diagnosis, % | 100.0 |
| No beta-blocker medication, % | 28.2 |
| No ACE inhibitor medication, % | 13.2 |
| Myocardial infarction, % | 51.8 |
| Chronic atrial fibrillation, % | 17.8 |
| ACE, angiotensin-converting enzyme; BMI, body mass index; COPD, chronic obstructive pulmonary disease; EF, ejection fraction; NYHA, New York Heart Association; SBP, systolic blood pressure. | |

Table S18 – Baseline patient and disease characteristics of the model population for subgroup 18 (recent diagnosis: no)

|  | Baseline characteristics of the starting population |
| --- | --- |
| Sample size | 239 |
| Ejection fraction (EF), % (mean) | 25.12 |
| Age, years (mean) | 68.15 |
| Systolic blood pressure (SBP), mm Hg (mean) | 112.70 |
| Body mass index (BMI), kg/m2 (mean) | 26.67 |
| Creatinine, µmol/l (mean) | 144.87 |
| NYHA class 1, % | 15.2 |
| NYHA class 2, % | 42.0 |
| NYHA class 3, % | 34.5 |
| NYHA class 4, % | 8.3 |
| Gender (male), % | 76.4 |
| Smoker, % | 8.4 |
| Diabetes, % | 34.9 |
| Chronic obstructive pulmonary disease (COPD), % | 27.5 |
| Recent diagnosis, % | 0.0 |
| No beta-blocker medication, % | 44.6 |
| No ACE inhibitor medication, % | 20.5 |
| Myocardial infarction, % | 61.7 |
| Chronic atrial fibrillation, % | 29.9 |
| ACE, angiotensin-converting enzyme; BMI, body mass index; COPD, chronic obstructive pulmonary disease; EF, ejection fraction; NYHA, New York Heart Association; SBP, systolic blood pressure. | |

Table S19 – Baseline patient and disease characteristics of the model population for subgroup 19 (no beta-blocker medication: yes)

|  | Baseline characteristics of the starting population |
| --- | --- |
| Sample size | 159 |
| Ejection fraction (EF), % (mean) | 25.00 |
| Age, years (mean) | 67.91 |
| Systolic blood pressure (SBP), mm Hg (mean) | 110.04 |
| Body mass index (BMI), kg/m2 (mean) | 25.73 |
| Creatinine, µmol/l (mean) | 141.14 |
| NYHA class 1, % | 15.5 |
| NYHA class 2, % | 37.8 |
| NYHA class 3, % | 35.2 |
| NYHA class 4, % | 11.5 |
| Gender (male), % | 81.3 |
| Smoker, % | 8.8 |
| Diabetes, % | 30.6 |
| Chronic obstructive pulmonary disease (COPD), % | 36.2 |
| Recent diagnosis, % | 33.0 |
| No beta-blocker medication, % | 100.0 |
| No ACE inhibitor medication, % | 27.3 |
| Myocardial infarction, % | 52.8 |
| Chronic atrial fibrillation, % | 25.7 |
| ACE, angiotensin-converting enzyme; BMI, body mass index; COPD, chronic obstructive pulmonary disease; EF, ejection fraction; NYHA, New York Heart Association; SBP, systolic blood pressure. | |

Table S20 – Baseline patient and disease characteristics of the model population for subgroup 20 (no beta-blocker medication: no)

|  | Baseline characteristics of the starting population |
| --- | --- |
| Sample size | 267 |
| Ejection fraction (EF), % (mean) | 24.84 |
| Age, years (mean) | 67.10 |
| Systolic blood pressure (SBP), mm Hg (mean) | 116.77 |
| Body mass index (BMI), kg/m2 (mean) | 26.47 |
| Creatinine, µmol/l (mean) | 134.40 |
| NYHA class 1, % | 22.0 |
| NYHA class 2, % | 45.9 |
| NYHA class 3, % | 28.6 |
| NYHA class 4, % | 3.5 |
| Gender (male), % | 75.9 |
| Smoker, % | 14.3 |
| Diabetes, % | 37.2 |
| Chronic obstructive pulmonary disease (COPD), % | 17.2 |
| Recent diagnosis, % | 50.7 |
| No beta-blocker medication, % | 0.0 |
| No ACE inhibitor medication, % | 12.4 |
| Myocardial infarction, % | 58.4 |
| Chronic atrial fibrillation, % | 28.3 |
| ACE, angiotensin-converting enzyme; BMI, body mass index; COPD, chronic obstructive pulmonary disease; EF, ejection fraction; NYHA, New York Heart Association; SBP, systolic blood pressure. | |

Table S21 – Baseline patient and disease characteristics of the model population for subgroup 21 (no ACE inhibitor medication: yes)

|  | Baseline characteristics of the starting population |
| --- | --- |
| Sample size | 79 |
| Ejection fraction (EF), % (mean) | 26.20 |
| Age, years (mean) | 69.50 |
| Systolic blood pressure (SBP), mm Hg (mean) | 114.06 |
| Body mass index (BMI), kg/m2 (mean) | 25.74 |
| Creatinine, µmol/l (mean) | 161.21 |
| NYHA class 1, % | 3.8 |
| NYHA class 2, % | 43.5 |
| NYHA class 3, % | 37.1 |
| NYHA class 4, % | 15.6 |
| Gender (male), % | 77.6 |
| Smoker, % | 8.6 |
| Diabetes, % | 30.4 |
| Chronic obstructive pulmonary disease (COPD), % | 27.8 |
| Recent diagnosis, % | 33.1 |
| No beta-blocker medication, % | 61.5 |
| No ACE inhibitor medication, % | 100.0 |
| Myocardial infarction, % | 58.0 |
| Chronic atrial fibrillation, % | 28.8 |
| ACE, angiotensin-converting enzyme; BMI, body mass index; COPD, chronic obstructive pulmonary disease; EF, ejection fraction; NYHA, New York Heart Association; SBP, systolic blood pressure. | |

Table S22 – Baseline patient and disease characteristics of the model population for subgroup 22 (no ACE inhibitor medication: no)

|  | Baseline characteristics of the starting population |
| --- | --- |
| Sample size | 347 |
| Ejection fraction (EF), % (mean) | 25.14 |
| Age, years (mean) | 66.94 |
| Systolic blood pressure (SBP), mm Hg (mean) | 114.91 |
| Body mass index (BMI), kg/m2 (mean) | 26.22 |
| Creatinine, µmol/l (mean) | 131.33 |
| NYHA class 1, % | 21.5 |
| NYHA class 2, % | 43.6 |
| NYHA class 3, % | 29.5 |
| NYHA class 4, % | 5.4 |
| Gender (male), % | 75.8 |
| Smoker, % | 13.6 |
| Diabetes, % | 35.9 |
| Chronic obstructive pulmonary disease (COPD), % | 24.1 |
| Recent diagnosis, % | 47.9 |
| No beta-blocker medication, % | 34.2 |
| No ACE inhibitor medication, % | 0.0 |
| Myocardial infarction, % | 56.1 |
| Chronic atrial fibrillation, % | 26.4 |
| ACE, angiotensin-converting enzyme; BMI, body mass index; COPD, chronic obstructive pulmonary disease; EF, ejection fraction; NYHA, New York Heart Association; SBP, systolic blood pressure. | |

Table S23 – Baseline patient and disease characteristics of the model population for subgroup 23 (myocardial infarction: yes)

|  | Baseline characteristics of the starting population |
| --- | --- |
| Sample size | 242 |
| Ejection fraction (EF), % (mean) | 25.00 |
| Age, years (mean) | 69.34 |
| Systolic blood pressure (SBP), mm Hg (mean) | 113.98 |
| Body mass index (BMI), kg/m2 (mean) | 25.99 |
| Creatinine, µmol/l (mean) | 140.40 |
| NYHA class 1, % | 21.0 |
| NYHA class 2, % | 43.6 |
| NYHA class 3, % | 26.7 |
| NYHA class 4, % | 8.7 |
| Gender (male), % | 81.5 |
| Smoker, % | 8.9 |
| Diabetes, % | 41.4 |
| Chronic obstructive pulmonary disease (COPD), % | 22.7 |
| Recent diagnosis, % | 39.0 |
| No beta-blocker medication, % | 33.5 |
| No ACE inhibitor medication, % | 18.2 |
| Myocardial infarction, % | 100.0 |
| Chronic atrial fibrillation, % | 22.0 |
| ACE, angiotensin-converting enzyme; BMI, body mass index; COPD, chronic obstructive pulmonary disease; EF, ejection fraction; NYHA, New York Heart Association; SBP, systolic blood pressure. | |

Table S24 – Baseline patient and disease characteristics of the model population for subgroup 24 (myocardial infarction: no)

|  | Baseline characteristics of the starting population |
| --- | --- |
| Sample size | 184 |
| Ejection fraction (EF), % (mean) | 23.99 |
| Age, years (mean) | 64.56 |
| Systolic blood pressure (SBP), mm Hg (mean) | 113.38 |
| Body mass index (BMI), kg/m2 (mean) | 26.20 |
| Creatinine, µmol/l (mean) | 128.43 |
| NYHA class 1, % | 17.1 |
| NYHA class 2, % | 46.0 |
| NYHA class 3, % | 32.6 |
| NYHA class 4, % | 4.3 |
| Gender (male), % | 70.1 |
| Smoker, % | 16.2 |
| Diabetes, % | 25.0 |
| Chronic obstructive pulmonary disease (COPD), % | 27.9 |
| Recent diagnosis, % | 48.0 |
| No beta-blocker medication, % | 40.8 |
| No ACE inhibitor medication, % | 16.9 |
| Myocardial infarction, % | 0.0 |
| Chronic atrial fibrillation, % | 31.4 |
| ACE, angiotensin-converting enzyme; BMI, body mass index; COPD, chronic obstructive pulmonary disease; EF, ejection fraction; NYHA, New York Heart Association; SBP, systolic blood pressure. | |

Table S25 – Baseline patient and disease characteristics of the model population for subgroup 25 (chronic atrial fibrillation: yes)

|  | Baseline characteristics of the starting population |
| --- | --- |
| Sample size | 112 |
| Ejection fraction (EF), % (mean) | 25.40 |
| Age, years (mean) | 72.32 |
| Systolic blood pressure (SBP), mm Hg (mean) | 116.44 |
| Body mass index (BMI), kg/m2 (mean) | 25.38 |
| Creatinine, µmol/l (mean) | 152.22 |
| NYHA class 1, % | 12.3 |
| NYHA class 2, % | 44.9 |
| NYHA class 3, % | 33.8 |
| NYHA class 4, % | 9.0 |
| Gender (male), % | 83.3 |
| Smoker, % | 6.8 |
| Diabetes, % | 38.6 |
| Chronic obstructive pulmonary disease (COPD), % | 24.7 |
| Recent diagnosis, % | 31.6 |
| No beta-blocker medication, % | 35.6 |
| No ACE inhibitor medication, % | 21.5 |
| Myocardial infarction, % | 44.9 |
| Chronic atrial fibrillation, % | 100.0 |
| ACE, angiotensin-converting enzyme; BMI, body mass index; COPD, chronic obstructive pulmonary disease; EF, ejection fraction; NYHA, New York Heart Association; SBP, systolic blood pressure. | |

Table S26 – Baseline patient and disease characteristics of the model population for subgroup 26 (chronic atrial fibrillation: no)

|  | Baseline characteristics of the starting population |
| --- | --- |
| Sample size | 314 |
| Ejection fraction (EF), % (mean) | 24.88 |
| Age, years (mean) | 65.87 |
| Systolic blood pressure (SBP), mm Hg (mean) | 112.78 |
| Body mass index (BMI), kg/m2 (mean) | 26.17 |
| Creatinine, µmol/l (mean) | 128.55 |
| NYHA class 1, % | 19.0 |
| NYHA class 2, % | 42.7 |
| NYHA class 3, % | 32.3 |
| NYHA class 4, % | 6.0 |
| Gender (male), % | 76.0 |
| Smoker, % | 14.5 |
| Diabetes, % | 35.2 |
| Chronic obstructive pulmonary disease (COPD), % | 23.5 |
| Recent diagnosis, % | 49.2 |
| No beta-blocker medication, % | 35.5 |
| No ACE inhibitor medication, % | 17.8 |
| Myocardial infarction, % | 58.0 |
| Chronic atrial fibrillation, % | 0.0 |
| ACE, angiotensin-converting enzyme; BMI, body mass index; COPD, chronic obstructive pulmonary disease; EF, ejection fraction; NYHA, New York Heart Association; SBP, systolic blood pressure. | |
